# Bile acids induce hepatic differentiation of mesenchymal stem cells

**DOI:** 10.1038/srep13320

**Published:** 2015-08-25

**Authors:** Iris Sawitza, Claus Kordes, Silke Götze, Diran Herebian, Dieter Häussinger

**Affiliations:** 1 Clinic of Gastroenterology, Hepatology and Infectious Diseases; 2 Department of General Pediatrics, Neonatology and Pediatric Cardiology, Heinrich Heine University, Moorenstraße 5, 40225 Düsseldorf, Germany

## Abstract

Mesenchymal stem cells (MSC) have the potential to differentiate into multiple cell lineages and their therapeutic potential has become obvious. In the liver, MSC are represented by stellate cells which have the potential to differentiate into hepatocytes after stimulation with growth factors. Since bile acids can promote liver regeneration, their influence on liver-resident and bone marrow-derived MSC was investigated. Physiological concentrations of bile acids such as tauroursodeoxycholic acid were able to initiate hepatic differentiation of MSC via the farnesoid X receptor and transmembrane G-protein-coupled bile acid receptor 5 as investigated with knockout mice. Notch, hedgehog, transforming growth factor-β/bone morphogenic protein family and non-canonical Wnt signalling were also essential for bile acid-mediated differentiation, whereas β-catenin-dependent Wnt signalling was able to attenuate this process. Our findings reveal bile acid-mediated signalling as an alternative way to induce hepatic differentiaion of stem cells and highlight bile acids as important signalling molecules during liver regeneration.

Stellate cells are retinoid-storing cells with long cellular processes, which occur in several organs. In the liver, stellate cells store exceptionally high amounts of retinoids mainly as retinyl palmitate in prominent membrane-coated lipid droplets. Retinoids preserve the quiescent state of hepatic stellate cells (HSC)^1^,^2^ and are lost when the cells activate and develop into proliferating myofibroblast-like cells, which are capable to produce extracellular matrix proteins. Research on stellate cells mainly focussed on this process, since HSC can contribute to fibrogenesis and scar formation in chronic liver diseases^3^ and therapeutic approaches to treat fibrosis are urgently needed. However, little is known about the function of HSC in normal liver and also their identity remained mysterious for long time, because HSC express mesenchymal and neuroectodermal genes at the same time^4^,^5^,^6^,^7^. It was discovered recently that HSC are liver-resident MSC as evidenced by their MSC-related expression pattern and functional analyses^8^,^9^, which can derive from or home in the bone marrow^10^,^11^,^12^. Lineage tracing and transplantation studies revealed that stellate cells are capable to contribute to liver regeneration through differentiation into epithelial cell lineages such as hepatocytes and cholangiocytes^12^,^13^,^14^,^15^,^16^ as reported for MSC from the bone marrow or adipose tissue^17^,^18^,^19^. This direct contribution of MSC to liver repair is still controversially discussed. However, growth factor treatment of isolated stellate cells from rat liver and pancreas as well as MSC from the bone marrow (bmMSC) and umbilical cord blood (UCBSC) can initiate their differentiation into hepatocytes also *in vitro*^12^,^14^,^20^. These findings underline the potential of MSC from various sources to generate hepatocytes. During growth factor-mediated hepatic differentiation, all MSC populations transiently exhibit the typical expression pattern of liver progenitor cells with hepatobiliary characteristics, which are called oval cells in rodents, indicating that MSC and established liver progenitor cells are related cell types and can contribute to liver regeneration^12^.

Bile acids were recently not only recognized as important regulators of metabolism^21^,^22^,^23^,^24^,^25^, but also as mediators of liver regeneration, since cholic acid can promote liver regeneration after partial hepatectomy (PHX) in mice^26^. In contrast to this, high bile acid concentrations in cholestatic liver disease have adverse effects by inducing apoptosis and necrosis of hepatocytes. Interestingly, HSC are resistant against bile acid-mediated apoptosis and show increased cell proliferation in response to elevated bile acid concentrations^27^,^28^ and promote the development of fibrosis in cholestatic liver disease^27^. However, ligands of the nuclear farnesoid X receptor (Fxr) such as 6-ethyl chenodeoxycholic acid decreased also the production of extracellular matrix in cultured HSC and animal models of liver fibrosis^29^. Also the transmembrane G-protein-coupled bile acid receptor 5 (Tgr5) is expressed by HSC, but only when they are activated in culture^30^. The relevance of Tgr5 in activated stellate cells remained unknown thus far. Since HSC are obviously targets of bile acids and Fxr signalling was supposed to control the differentiation of bmMSC into osteoblasts^31^, we investigated, whether bile acids can influence developmental fate decisions of MSC from liver, bone marrow and umbilical cord blood. It turned out that physiological concentrations of bile acids can trigger the differentiation of rodent and human MSC into hepatocyte-like cells *in vitro*, which involves the bile acid receptors Fxr and Tgr5 as well as several signalling pathways known to control stem cell development.

## Results

**Figure 1:**
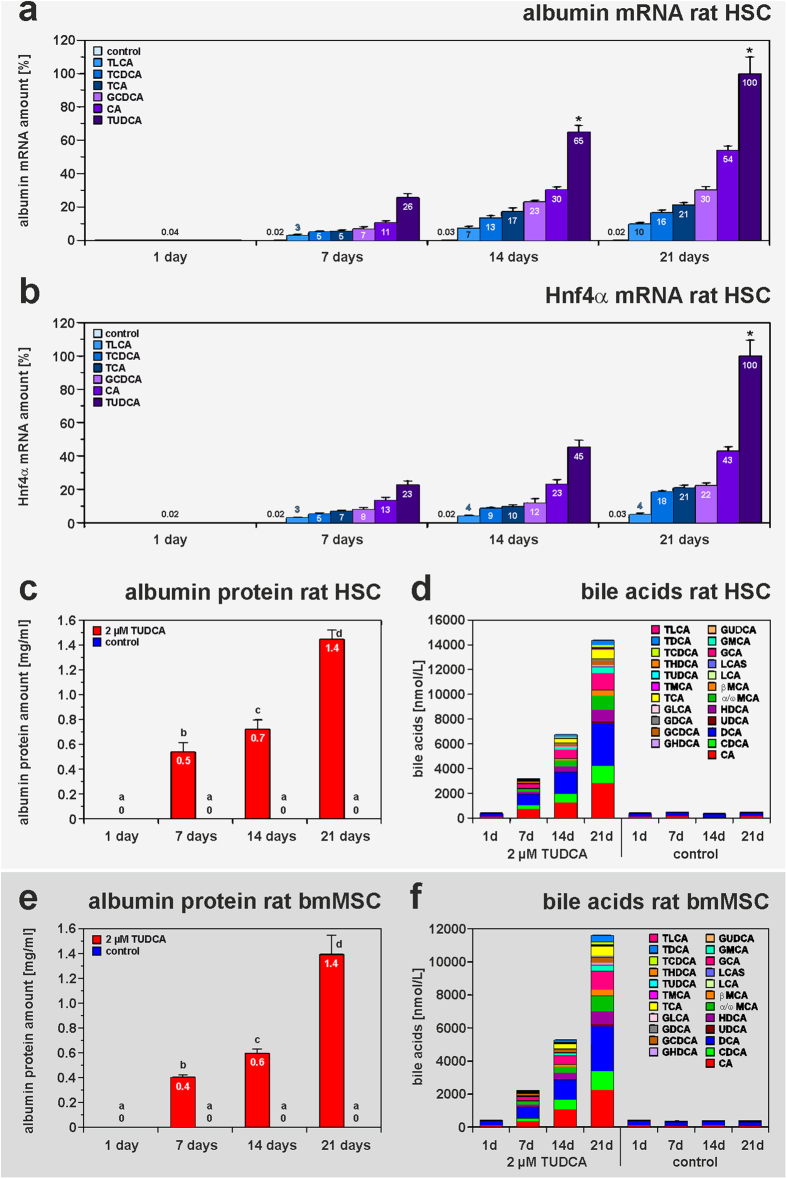
Bile acids promote hepatic differentiation of HSC and bmMSC from rats. (**a,b**) Freshly isolated rat HSCs were treated with 2 μM TLCA, TCDCA, TCA, GCDCA, CA or TUDCA for 21 days in serum-free media (n = 3). The HSCs were analysed in weekly intervals by qPCR of (**a**) albumin and (**b**) Hnf4α mRNA. Hprt1 expression was used for the normalization of the values and the highest mRNA amount of the hepatic markers measured by qPCR was set to 100%. The gradual increase of the hepatocyte markers albumin and Hnf4α in HSC cultures indicated that stellate cells differentiated into liver parenchymal cells in response to bile acid treatment. In contrast to this, HSC of the control, which were treated with serum-free medium without bile acids, remained negative for hepatocyte markers. (**c,d**) Freshly isolated HSC and (**e,f**) cultured bmMSC from rats were treated with 2 μM TUDCA for 21 days (n = 3). (**c,e**) The hepatocyte marker albumin was quantified at the protein level by ELISA in culture supernatants. (**d,f**) The release of bile acids into the culture medium was measured by UHPLC-MS/MS in weekly intervals. Hepatic differentiation as indicated by induced albumin expression and bile acid synthesis was congruently initiated in rat HSC and bmMSC of rats by TUDCA treatment, whereas MSC populations without TUDCA (control) remained negative for these hepatocyte markers. TUDCA levels close to and above 2 μmol/L were also measured by UHPLC-MS/MS. Since TUDCA was added to the medium, it was not included in the graphs. (**d,f**) Small amounts of bile acids were already found in the culture medium by UHPLC-MS/MS before incubation of cells, which may account for the low concentrations of bile acids measured at day 1 and under control conditions.

**Figure 2:**
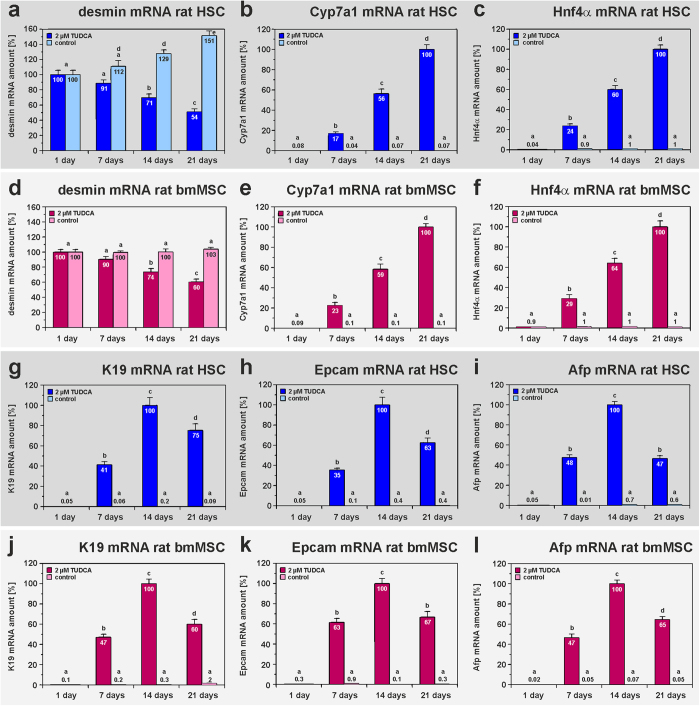
Intermediate states of mesenchymal and epithelial cells appear in TUDCA-treated HSC and bmMSC from rats during hepatic differentiation. (**a–c**,**g–i**) Freshly isolated HSC and (**d–f**,**j–l**) cultured bmMSC from rats were treated with 2 μM TUDCA for 21 days. The progression of cell differentiation was analysed in weekly intervals by qPCR (n = 3). After initiation of hepatic differentiation by TUDCA, HSC and bmMSC decreased their (**a,d**) desmin expression and started to express the hepatocyte markers (**b,e**) Cyp7a1 and (**c,f**) Hnf4α. The transient appearance of (**g,j**) K19, (**h,k**) Epcam and (**i,l**) Afp, indicated that HSC and bmMSC first become epithelial progenitor cells before they develop into hepatocyte-like cells.

The concentration of many bile acids in the blood serum of rats transiently increased after liver injury by partial hepatectomy (PHX) (supplemental Fig. S1; supplemental Table S1, S2). The finding that bile acids can support the regeneration of injured liver tissue^26^ prompted us to investigate, whether bile acids can influence the hepatic differentiation of MSC from liver (hepatic stellate cells, HSC), bone marrow (bmMSC) and umbilical cord blood (UCBSC) from rodents and bmMSC from humans (hbmMSC). Various bile acids such as GCDCA (glycochenodeoxycholic acid), TUDCA (tauroursodeoxycholic acid), CA (cholic acid), GCDCA (glycochenodeoxycholic acid), TCA (taurocholic acid), TCDCA (taurochenodeoxycholic acid), and TLCA (taurolithocholic acid) were used and triggered the differentiation of HSC into hepatocyte-like cells *in vitro*. Primary cultures of rat HSC were treated with 2 μM of individual bile acids dissolved in a serum-free medium for 21 days. The initiation of hepatic differentiation of HSC was indicated by the induction of the hepatocyte markers albumin and hepatocyte nuclear factor 4α (Hnf4α) as measured by quantitative reverse-transcriptase polymerase chain reaction (qPCR) in adherent cells (Fig. 1a,b). Among the bile acids tested, TUDCA was found to be most potent. TUDCA was also tested at lower (0.5 μM and 1 μM) and higher (20 μM and 100 μM) concentrations on primary cultures of HSC. Compared to 2 μM TUDCA, the other concentrations of TUDCA led to lower albumin expression, indicating insufficient hepatic differentiation or loss of cells with time (not shown). Therefore, 2 μM TUDCA were used in the following experiments. TUDCA-mediated induction of hepatic differentiation was not only detectable at the mRNA level but also by the release of albumin protein (Fig. 1c; supplemental Fig. S2) and the wide spectrum of bile acids (Fig. 1d) secreted into the culture medium as analysed by an enzyme-linked immunosorbent assay (ELISA) specific for rat albumin and ultra-high-performance liquid chromatography–tandem mass spectrometry (UHPLC-MS/MS), respectively. The initiation of albumin and bile acid production indicated that HSC acquired functional characteristics of hepatocytes after TUDCA application in the absence of growth factor treatment. Since the whole culture medium was changed every second to third day, the values reflected increased synthesis of albumin and bile acids of the cells with time in culture. TUDCA-triggered hepatic differentiation was not restricted to HSC, but was also found in cultured rat bmMSC as indicated by the induction of albumin, Hnf4α and cytochrome P450 7A1 (Cyp7a1) expression as well as by a bile acid production, which constantly increased with time (Figs 1e,f and 2a–f). In contrast to this, HSC and bmMSC of the control, which received the same medium but without TUDCA, showed no hints of hepatic differentiation (Figs 1a–f and 2a–f). Also clonally expanded HSC and UCBSC from rats with characteristics of MSC were able to differentiate into hepatocyte-like cells *in vitro* in response to TUDCA treatment (supplemental Tables S3-S7). This effect was obviously limited to stem cells such as MSC, since fibroblasts from the abdominal muscle of rats did not differentiate into hepatocytes within 21 days of TUDCA treatment (supplemental Tables S3-S7). During TUDCA-mediated differentiation the HSC reached approximately 23% of the albumin mRNA expression found in cultured hepatocytes (supplemental Table S3). The mRNA levels of Cyp7a1 and Hnf4α reached 12% and 10% of isolated hepatocytes, respectively (supplemental Table S6, S7). The initiation of hepatic differentiation by bile acids was not restricted to rodent MSC. Also hbmMSC, which express typical MSC markers such as vimentin and platelet-derived growth factor receptor β (PDGFRβ), showed hepatic differentiation in response to TUDCA treatment as indicated by the induction of albumin, sodium-taurocholate-cotransporting polypeptide (NTCP) and HNF4α (supplemental Fig. S3).

**Figure 3:**
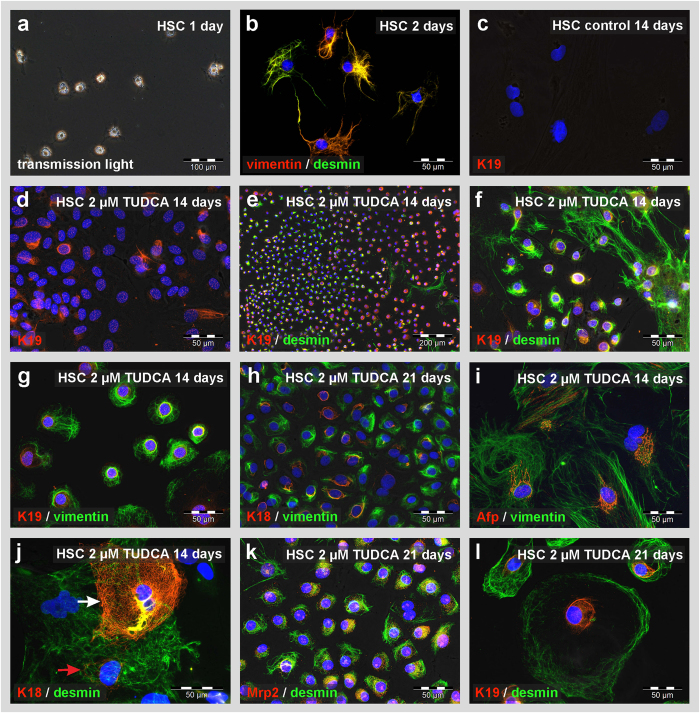
TUDCA induces intermediate states of mesenchymal and epithelial cells in primary cultures of rat HSC during hepatic differentiation. Freshly isolated rat HSC (**a**) exhibited typical lipid droplets that contained retinoids and (**b**) expressed the mesenchymal filament proteins vimentin (red) and desmin (green) as investigated by immunofluorescence. (**c**) Under control conditions HSC remained negative for K19 expression, (**d**) but this epithelial marker protein was induced in HSC cultures after treatment with 2 μM TUDCA for 14 days (red). (**e–g**) K19 was found to be co-expressed with desmin and vimentin (green), which indicated the origin of epithelial progenitor cells from HSC. (**h–k**) Also K18, Afp and Mrp2 (red), which served as markers for hepatic differentiation, were co-expressed with vimentin and desmin (green) after 21 days of TUDCA treatment. Cells with different states of maturation were still found after 21 days of TUDCA treatment. Some cells developed into hepatocyte-like cells with (**j**) dominant K18 filaments (white arrows) while others remained immature with persisting desmin (red arrow) or (**l**) K19 protein residues.

TUDCA-triggered hepatic differentiation of MSC from the rat liver and bone marrow was accompanied by a decreased expression of mesodermal markers such as desmin and the transient acquisition of the expression profile of hepatic progenitor cells (oval cells) before they differentiate into hepatocyte-like cells as investigated by qPCR (Fig. 2a–l). Such hepatic progenitor cells are characterized by the expression of keratin 19 (K19), epithelial cell adhesion molecule (Epcam) and α-fetoprotein (Afp). The hepatocyte markers albumin, Cyp7a1 and Hnf4α steadily increased during TUDCA treatment, indicating progression of hepatic differentiation of MSC (Fig. 2a–l). A similar transient increase of progenitor cell markers was also found when HSC clones and UCBSC were treated with TUDCA, but muscle fibroblasts remained negative for these markers (supplemental Table S8-S11). The formation of hepatic progenitor cells and hepatocyte-like cells by HSC was confirmed by immunofluorescence staining (Fig. 3). Freshly isolated HSC with prominent lipid droplets and mesodermal filament proteins desmin and vimentin (Fig. 3a,b), started to co-express epithelial markers such as K18, K19, Afp and multidrug resistance protein 2 (Mrp2) after 14 and 21 days of TUDCA treatment (Fig. 3d–l), while HSC of the control remained negative for epithelial markers such as K19 (Fig. 3c). Although residual desmin and vimentin filaments were still found, more mature hepatocyte-like cells without mesodermal markers were also detected by immunofluorescence staining at day 21 of TUDCA treatment (Fig. 3j).

**Figure 4:**
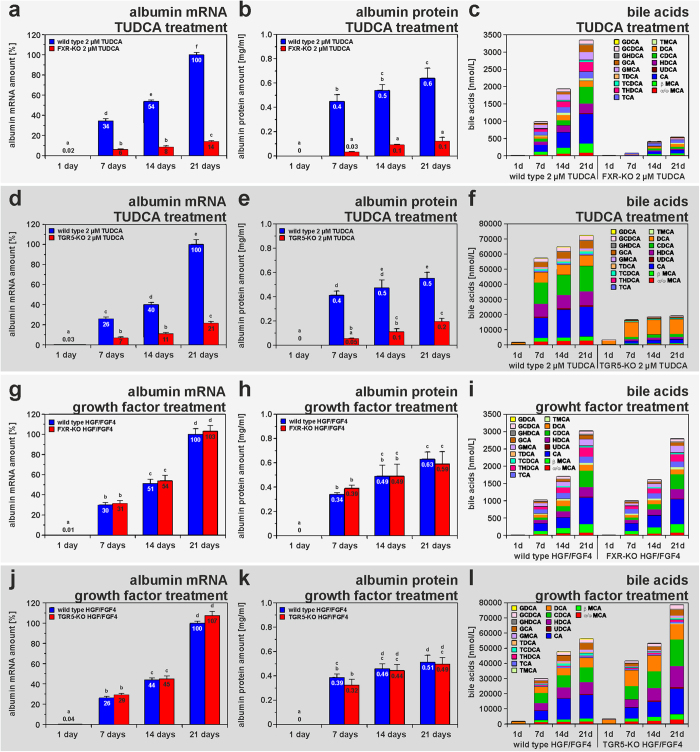
TUDCA but not growth factors promote hepatic differentiation via Fxr and Tgr5 in bmMSC from mice. Cultured bmMSC from FXR-KO and corresponding wild type control mice were either treated with (**a–c**) 2 μM TUDCA or (**g–i**) growth factors (40 ng/ml HGF, 50 ng/ml FGF4) for 21 days (n = 3). The expression of (**a,g**) albumin was analysed by qPCR in weekly intervals. The release of (**b,h**) albumin and (**c,i**) bile acids was measured by ELISA and UHPLC-MS/MS at indicated time points. The bmMSC from Tgr5-KO and wild type control mice were also treated with (**d–f**) 2 μM TUDCA or (**j–l**) growth factors and analysed in a similar way (n = 3). The strong suppression of albumin expression and bile acid synthesis in bmMSC cultures from (**a–c**) Fxr-KO and (**d–f**) Tgr5 mice indicated that both receptors are involved in bile acid-initiated hepatic differentiation, (**g–l**) whereas the growth factors induce the same process independent from bile acid receptors.

**Figure 5:**
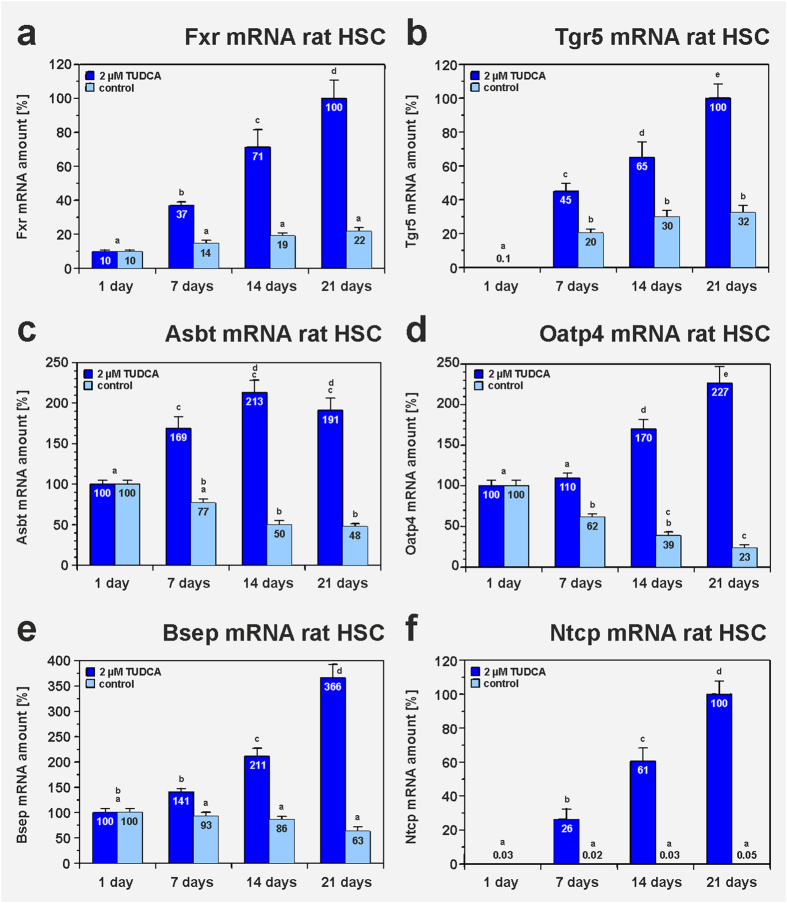
Expression of bile acid receptors and transporters in freshly isolated and TUDCA-treated HSC from rats. Freshly isolated HSC from rats were treated with 2 μM TUDCA for 21 days and the expression of the bile acid receptors (**a**) Fxr and (**b**) Tgr5 as well as the bile acid transporters (**c**) Asbt, (**d**) Oatp4, (**e**) Bsep and (**f**) Ntcp was investigated by qPCR in weekly intervals (n = 3). HSC of the control received the same medium but without TUDCA. The expression of (**a**) Fxr, (**c**) Asbt, (**d**) Oatp4 and (**e**) Bsep mRNA was already detectable in freshly isolated HSC (day 1) but increased significantly in response to TUDCA treatment. (**b**) The mRNA of Tgr5 remained undetectable in freshly isolated HSC but appeared during culture of HSC under control conditions and after TUDCA treatment. (**f**) Ntcp expression was not found in freshly isolated HSC and under control conditions, but was induced in response to TUDCA administration and served as an additional marker for hepatic differentiation.

**Figure 6:**
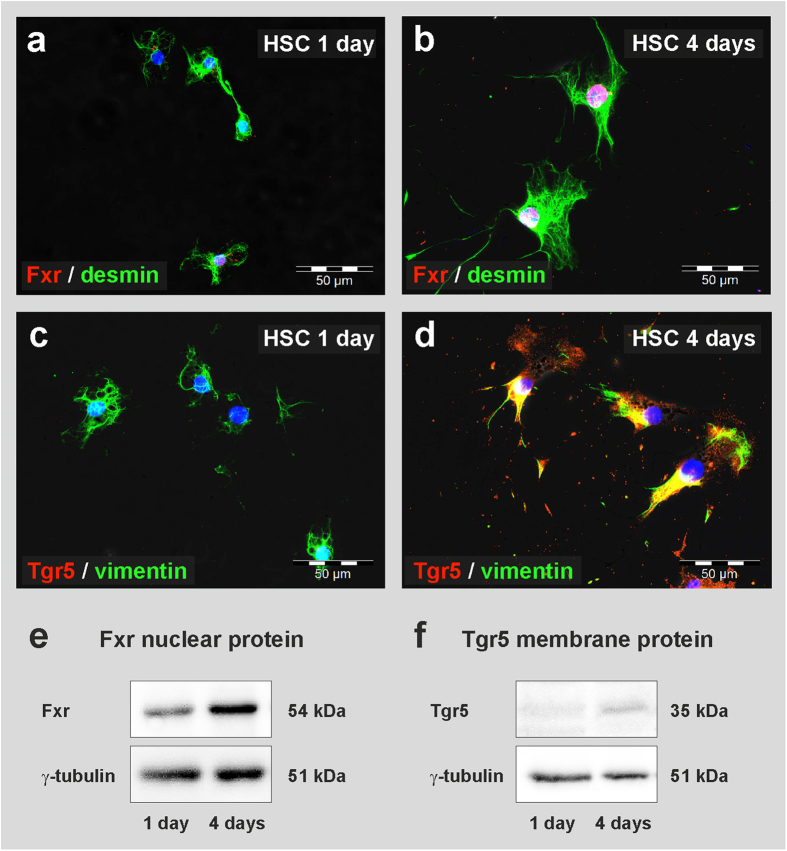
Detection of bile acid receptors in freshly isolated and activated HSC from rats. Freshly isolated (1 day) and cultured (4 days) HSC were stained with antibodies against the bile acid receptors (**a,b**) Fxr and (**c,d**) Tgr5 (red). The mesenchymal markers desmin and vimentin (green) were used to verify the presence of stellate cells. HSC cultured for 4 days were maintained in medium without serum and without TUDCA. (**a**) Few freshly isolated HSC displayed weak nuclear staining of Fxr, but most cells lacked clear nuclear Fxr staining one day after isolation. (**b**) The majority of HSC exhibited significant nuclear Fxr after few days in culture. (**c**) Tgr5 was not detectable in freshly isolated HSC by immunofluorescence, (**d**) but appeared during culture within few days. (**e**) The presence of Fxr in freshly isolated and cultured HSC was confirmed by Western blot analysis. Fxr was detected in the nuclear protein faction mainly after 4 days of culture (10 μg protein per lane). (**f**) Tgr5 was found in cell membrane protein fraction of HSC after 4 days of culture (30 μg protein per lane). The protein γ-tubulin served as a control.

The molecular mechanisms underlying the TUDCA-mediated hepatic differentiation of MSC were investigated by the use of isolated bmMSC from Fxr and Tgr5 knockout (KO) mice. The knockout of Fxr and Tgr5, respectively, was verified by qPCR in comparison with bmMSC from the corresponding wild type mouse strains. Fxr and Tgr5 expression was only found in bmMSC from wild type control mice (not shown). The deletion of either Fxr (Fig. 4a–c) or Tgr5 (Fig. 4d–f) significantly inhibited the TUDCA-mediated hepatic differentiation of bmMSC as indicated by decreased albumin expression and bile acid production when compared to the results obtained with bmMSC from wild type mouse strains. This suggests that both bile acid receptors are involved in the hepatic differentiation induced by bile acids. The requirement of Tgr5 for the initiation of this process was also observed in primary cultures of isolated HSC from Tgr5-KO mice. HSC without Tgr5 showed no significant differentiation after TUDCA treatment, whereas HSC from wild type mice developed into hepatocyte-like cells as indicated by their albumin release (supplemental Fig. S4). However, growth factor-mediated hepatic differentiation was found to be independent from bile acid receptors, since the differentiation of MSC into hepatocyte-like cells was unaffected in bmMSC from Fxr and Tgr5 knockout mice during treatment with hepatocyte growth factor (HGF) and fibroblast growth factor 4 (FGF_4_) (Fig. 4g–l). The expression of Fxr and Tgr5 was found to be up-regulated early during culture of rat HSC as investigated by qPCR (Fig. 5a,b), immunofluorescence and Western blot analysis (Fig. 6a–f). After 4 days in culture, Fxr became clearly detectable in the cell nuclei and Tgr5 appeared in the cell membrane of rat HSC under serum-free conditions (Fig. 6a–f), indicating that only activated HSC can properly respond to bile acids. The expression of Fxr and Tgr5 strongly increased in HSC when TUDCA was added to the culture medium (Fig. 5a,b). Also bile acid transporters such as the apical sodium-dependent bile acid transporter (Asbt), organic anion-transporting polypeptide 4 (Oatp4) and bile salt export pump (Bsep) were stronger expressed in response to TUDCA treatment (Fig. 5c–e, supplemental Fig. S5a–f). Although Asbt and Oatp4 were found to be expressed in freshly isolated HSC as investigated at the mRNA level, only small amounts of these transporters were detectable at protein level by immunofluorescence staining (supplemental Fig. S5a–h), suggesting that quiescent HSC can take up bile acids at least in principle. The expression of Bsep remained weak and Ntcp was undetectable in freshly isolated rat HSC as analysed by qPCR (Fig. 5e,f). The hepatocyte-specific Ntcp remained undetectable in HSC without TUDCA treatment (control), but was induced after application of TUDCA, as another indication for their differentiation into hepatocyte-like cells (Fig. 5f; supplemental Fig. S5g, h).

**Figure 7:**
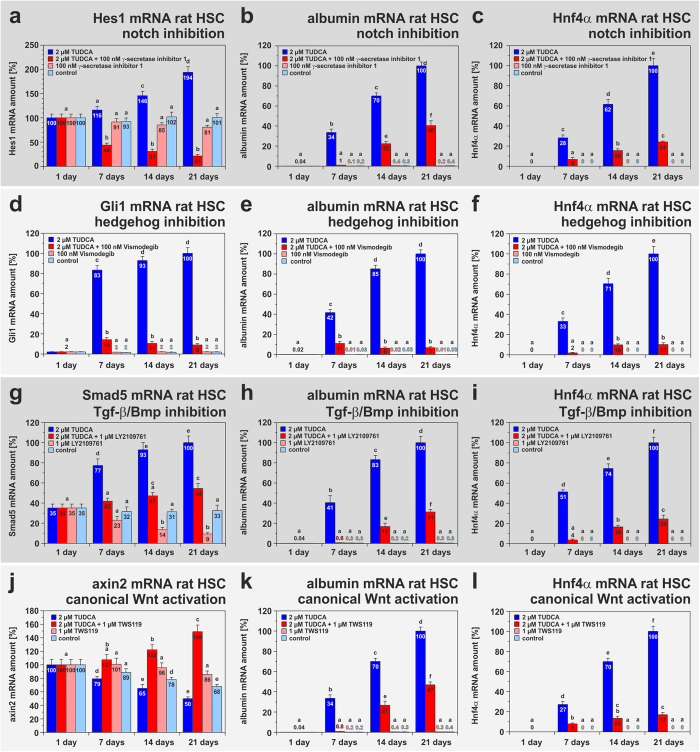
Notch, hedgehog, Tgf-β/Bmp and canonical Wnt signalling pathways control TUDCA-mediated hepatic differentiation of rat HSC. Freshly isolated HSC from rats were treated with either 2 μM TUDCA, small inhibitory molecules or a combination of these substances for 21 days. HSC of the control received medium without TUDCA and small inhibitory molecules. The expression of target genes of (**a**) notch (Hes1), (**d**) hedgehog (Gli1), (**g**) Tgf-β/Bmp (Smad5) and (**j**) canonical Wnt signalling (axin2) was investigated by qPCR in weekly intervals (n = 3–6). The influence of these signalling pathways on hepatic differentiation of HSC was investigated by the expression of the hepatocyte markers (**b,e,h,k**) albumin and (**c,f,i,l**) Hnf4α using qPCR. (**a–c**) Notch signalling was inhibited by 100 nM γ-secretase inhibitor 1, (**b,c**) which significantly blocked hepatic differentiation by TUDCA. Cell differentiation was also negatively affected when **(e,f**) hedgehog signalling was inhibited by 100 nM Vismodegib or **(h,i**) Tgf-β/Bmp signalling was inhibited by 1 μM LY2109761. TUDCA treatment obviously promoted notch, hedgehog and Tgf-β/Bmp signalling pathways as indicated by elevated (**a**) Hes1, (**d**) Gli1 and (**g**) Smad5 expression. (**j**) In contrast to this, axin2 expression as a target of canonical Wnt signalling was suppressed by TUDCA application. (**k,l**) In line with this, the inhibition of Gsk3β activity by 1 μM TWS119, which mimics canonical Wnt signalling, significantly prevented TUDCA-mediated hepatic differentiation of HSC.

Next we addressed the question whether signalling pathways known to guide developmental fate decisions of stem cells such as notch, hedgehog, transforming growth factor-β family/bone morphogenetic protein (Tgf-β/Bmp) and wingless type (Wnt) are also required for TUDCA-mediated hepatic differentiation of HSC. An integral part of notch signalling is the enzymatic cleavage of the intracellular domain of ligand-bound notch receptors by γ-secretase. This enzyme was inhibited in cultured HSC by 100 nM γ-secretase inhibitor 1 in the presence and absence of TUDCA. Treatment of HSC with TUDCA elevated the notch target gene hairy enhancer of split 1 (Hes1) (Fig. 7a) and was found to be associated with a general increase of notch signalling as indicated by up-regulation of notch3 receptor expression (supplemental Fig. S6). However, Hes1 expression decreased when TUDCA was combined with γ-secretase inhibitor (Fig. 7a). This inhibition of notch signalling reduced also hepatic differentiation as indicated by lowered albumin and Hnf4α expression (Fig. 7b,c; supplemental Fig. S7a). TUDCA-mediated hepatic differentiation of HSC was also associated with an increase of glioma-associated oncogene homolog 1 (Gli1) as well as Smad2 and Smad5 (vertebrate orthologs of mothers against decapentaplegic) expression (Fig. 7d,g; supplemental Fig. S8a), suggesting that hedgehog and Tgf-β/Bmp signalling are also enhanced during this process. The inhibition of hedgehog signalling by 100 nM Vismodegib and Tgf-β/Bmp signalling by 1 μM LY2109761 lowered not only the target gene expression but also the hepatic differentiation of HSC initiated by TUDCA (Fig. 7d–i; supplemental Fig. S7b,c). Interestingly, the expression of collagen type 1 α2 chain (Col1α2) was not down-regulated by the Tgf-β receptor I/II inhibitor LY2109761, whereas TUDCA treatment significantly prevented the increase of Col1α2 (supplemental Fig. S8b) and α-smooth mucle actin (α-Sma) expression in cultured HSC (not shown).

**Figure 8:**
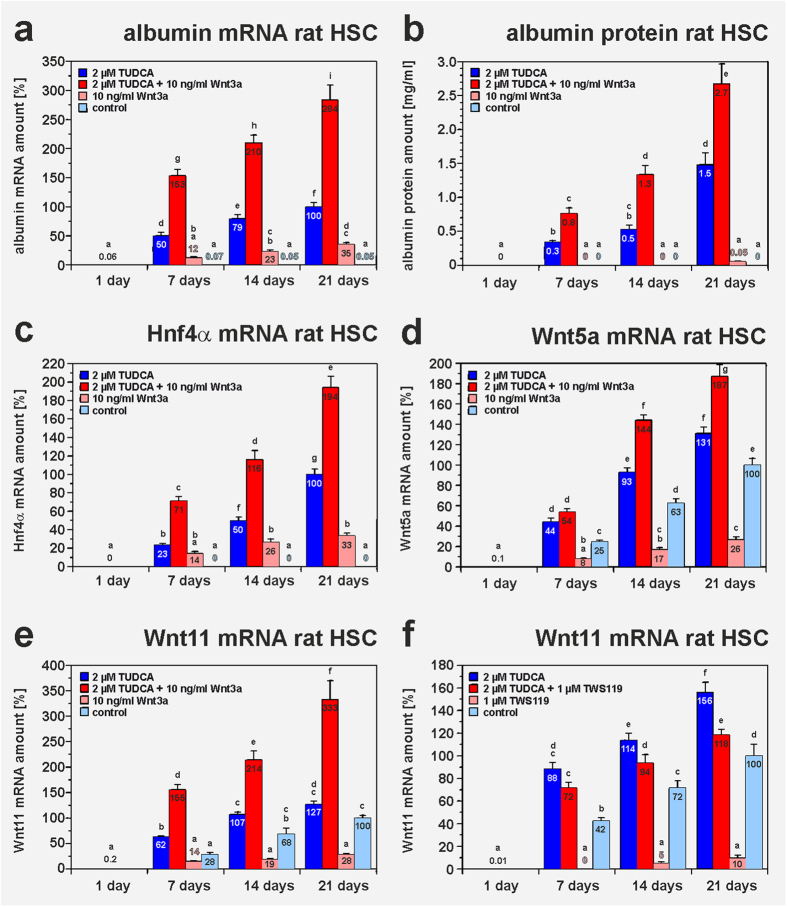
Wnt3a supports TUDCA-mediated hepatic differentiation of rat HSC via non-canonical Wnt signalling. Freshly isolated HSC from rats were treated with either 2 μM TUDCA, 10 ng/ml Wnt3a or a combination of both agents for 21 days. HSC of the control received medium without TUDCA and Wnt3a (n = 3–4). The expression of the hepatocyte markers albumin and Hnf4α as well as the non-canonical Wnt target genes Wnt5a and Wnt11 was analysed by qPCR and ELISA. Wnt3a had synergistic effects on TUDCA-mediated hepatic differentiation of HSC as indicated by significantly increased **(a,b**) albumin and (**c**) Hnf4α expression when both agents were combined. TUDCA alone and in combination with Wnt3a promoted non-canonical Wnt signalling as indicated by elevated (**d**) Wnt5a and (**e**) Wnt11 expression. (**f**) In contrast to this, canonical Wnt signalling mimicked by 1 μM TWS119 lowered the effect of TUDCA and had strong inhibitory effects on Wnt11 expression when the Gsk3β inhibitor was applied alone.

TUDCA-induced differentiation led to decreased axin2 expression in HSC, which is a target gene of β-catenin-dependent Wnt signalling (canonical Wnt) (Fig. 7j). Mimicking of canonical Wnt signalling by the glycogen synthase kinase 3β (Gsk3β) inhibitor TWS119 (1 μM) induced elevated axin2 levels (Fig. 7j), but diminished TUDCA-mediated hepatic differentiation as indicated by decreased albumin and Hnf4α expression (Fig. 7k,l; supplemental Fig. S7d). Similar results were also obtained when HSC were treated with the Gsk3β inhibitor BIO (supplemental Fig. S7e,f). Mimicking of the canonical Wnt signalling by TWS119 or BIO was found to be associated with a down-regulation of Fxr and Tgr5 expression (supplemental Fig. S9a,b). This could also explain the inhibitory effect of this signalling pathway on hepatic differentiation. Since evidence exists that Wnt3a supports the differentiation of progenitor cells into hepatocytes^32^, this Wnt ligand was applied alone and in combination with TUDCA on freshly isolated HSC. In fact, 10 ng/ml Wnt3a had synergistic effects to TUDCA on hepatic differentiation of HSC as indicated by the strong increase of albumin and Hnf4α expression, but Wnt3a alone exerted only mild effects (Fig. 8a–c). After simultaneous treatment of HSC with TUDCA and Wnt3a, the rat HSC reached about 58% of the albumin mRNA amount found in freshly isolated rat hepatocytes (supplemental Table S4). Although Wnt3a alone weakly induced canonical Wnt as suggested by lowered Wnt5a and Wnt11 expression, Wnt3a in combination with TUDCA induced the reverse by promoting Wnt5a and Wnt11 expression (Fig. 8d,e). This indicates that the effects of Wnt3a are dependent upon the signalling context and can support hepatic differentiation as well as non-canonical Wnt signalling in the presence of TUDCA.

## Discussion

Bile acids such as CA can support liver regeneration in rodents after PHX via an Fxr-dependent mechanism^26^. In fact, the blood concentrations of bile acids increase in rats after PHX and could potentially affect the behaviour of hepatocytes or stem cell populations, which both can contribute to the repair of injured liver. Until now, it remained unclear whether developmental fate decisions of adult stem cells are influenced by bile acids. We found that bile acids can initiate the hepatic differentiation of isolated MSC from liver, bone marrow and umbilical cord blood of rodents and humans. During this process, MSC transiently acquired the expression profile of liver progenitor cells as observed after treatment with the growth factors HGF and FGF4^12^, which are frequently used to induce the differentiation of MSC into hepatocytes. Triggering stem cell differentiation may contribute to beneficial effects of bile acids during liver repair^26^ and fibrosis^29^, since TUDCA-triggered hepatic differentiation of HSC was not only associated with an increase of hepatocyte markers but also with decreased Col1α2 and α-Sma expression.

Although TUDCA was found to be most potent among the bile acids tested, TUDCA was reported to be a weak ligand of Fxr and Tgr5^33^,^34^, suggesting that yet unknown mechanisms are probably involved to mediate this process via these bile acid receptors, which await further investigation. However, the wide spectrum of bile acids released by MSC in response to TUDCA most likely support Fxr and Tgr5-mediated cell differentiation at later time points. The low abundance of bile acid transporters and Tgr5 expression in freshly isolated HSC indicated that quiescent HSC must activate before they can properly respond to bile acid exposure. Indeed, Fxr protein levels were up-regulated and Tgr5 expression was induced during HSC activation within few days of culture. Both receptors are essential for bile acid-mediated hepatic differentiation of MSC as demonstrated by the use of Fxr and Tgr5 knockout mice. Interestingly, it was already shown that a stimulation of Fxr is also necessary for the differentiation of bmMSCs into osteoblasts^31^. On the one hand, Fxr seems to be required for MSC differentiation, but on the other hand, growth factor-mediated hepatic differentiation of MSC occurred independently from Fxr as investigated with bmMSC from Fxr knockout mice. Thus, bile acids apparently promote cell differentiation via alternative routes that differ from those adressed by growth factors.

TUDCA-mediated hepatic differentiation requires not only the presence of the bile acid receptors Fxr and Tgr5 but also notch, hedgehog and Tgf-β/Bmp signalling. Inhibition of these signalling cascades by small inhibitory molecules impaired proper cell differentiation of HSC, whereas promotion of canonical Wnt signalling was able to abolish this process. Maintenance of Wnt signalling via β-catenin by the Gsk3β inhibitors TWS119 or BIO counteracts HSC activation^35^ and represents an essential element of the stellate cell niche in the space of Disse^36^,^37^. As demonstrated in the present study, mimicking of canonical Wnt signalling lowered the expression of Fxr and Tgr5 by HSC. This mechanism could prevent unnecessary cell differentiation in the normal liver, because HSC are always exposed to low concentrations of bile acids through the blood serum. Since the expression of non-canonical Wnt signalling ligands such as Wnt5a and Wnt11 increase during HSC activation^35^ and both ligands are known to suppress canonical Wnt signalling and to promote the differentiation of progenitor cells^38^, we analysed their expression during TUDCA-mediated differentiation. While canonical Wnt signalling was suppressed by TUDCA as indicated by the lowered expression of the target gene axin2^39^, the expression of Wnt5a and Wnt11 was enhanced after addition of TUDCA. This indicates a pivotal role of non-canonical Wnt signalling in hepatic differentiation of MSC. The development of progenitor cells into hepatocytes during liver regeneration was found to be promoted by Wnt3a^32^. In fact, Wnt3a supported TUDCA-triggered hepatic differentiation of HSC, but this process was associated with an increase of Wnt5a and Wnt11. This indicates enhanced non-canonical Wnt signalling by Wnt3a in the presence of TUDCA. It is well known that the ligand Wnt3a can promote canonical and non-canonical Wnt pathways^39^. This dual role of Wnt3a was also visible in our experiments when the ligand was applied alone or in combination with TUDCA. Thus, the outcome of Wnt3a-mediated signalling appears to be context-dependent.

In conclusion, bile acids such as TUDCA can trigger hepatic differentiation of MSC from various sources. The bile acid receptors Fxr and Tgr5 are involved in this process, although their mode of action awaits further investigation. Since TUDCA-mediated cell differentiation was detectable in MSC from various sources of rodents and humans, natural occurring or modified bile acids may represent a novel tool to generate hepatocytes *in vitro*. This method could be used independently from growth factor treatment to facilitate the generation of patient-specific hepatocytes from stem cells for future therapeutic use.

## Methods

### Cell isolation and culture techniques

HSC were isolated from adult Wistar rats (>500 g) and Black 6 mice (>25 g), which were obtained from the animal facility of the Heinrich Heine University (Düsseldorf, Germany). Stellate cells were obtained by enzymatic digestion of the liver and density gradient centrifugation (8% Nycodenz, #1002424, Nycomed Pharma, Oslo, Norway) of the resulting non-parenchymal cell fraction essentially as described^40^. Isolated HSC were cultured in Dulbecco’s Modified Eagle Medium (DMEM, #41966-029, Gibco, Invitrogen, Karlsruhe, Germany) supplemented with 10% (rat) or 20% (mouse) fetal calf serum (FCS; Biochrom) and 1% antibiotic/antimycotic solution (#15240-062, Gibco) or under serum-free conditions as described below. HSC from rats were also maintained as single cell clones for several months as described^12^,^14^. UCBSC of unborn Wistar rats (18–20 days post coitum) were collected by flushing out the umbilical cord with culture medium and maintained as single cell clones for several months in Dulbecco’s Modified Eagle Medium Nutrient Mixture F-12 (DMEM/F12, #11330-032, Gibco) supplemented with 15% maintenance FCS (#06902, Stem Cell Technologies, Vancouver, Canada) and 1% antibiotic/antimycotic solution. MSC from the bone marrow of adult rats were obtained by rinsing the humeri with 0.5 mM ethylenediaminetetraacetic acid (EDTA; #8043.3, Carl Roth, Karlsruhe, Germany) dissolved in phosphate buffered saline supplemented with 2% FCS. Rat bone marrow cells were filtered, washed by centrifugation and blood cell lineages were depleted by using antibodies against CD3 (#MCA772F), CD45RA (#MCA340FT), CD161 (#MCA1427F, AbD Serotec, Oxford, UK) and CD11b (#SM1764F, Acris, Herford, Germany) coupled to fluorescein isothiocyanate (FITC) and the FITC EasySep selection Kit (#18558, Stem Cell Technologies). Bone marrow cells that remained unselected by this method were seeded on plastic dishes coated with collagen type 1 (#356401, BD Biosciences, Heidelberg, Germany), and cultured in MSC expansion medium (#CCM004; StemXVivo Mesenchymal Stem Cell Expansion Medium; R&D Systems, Minneapolis, MN, USA). A similar approach was used to enrich MSC from the humeri of Black 6 mice (wild type control) and knockout mouse strains for FXR (B6.129 × 1(FVB)-Nr1h4^tm1Gonz^/J) as well as TGR5 (C57BL/6.129-TgH-(Gpbar1)). The long bones of several mice were pestled in a mortar, collected in sterile buffer containing 0.5 mM EDTA, filtered and washed by centrifugation. Hematopoietic cell lineages were finally removed by magnetic cell sorting using the EasySep Mouse Mesenchymal Stem/Progenitor Cell Enrichment Kit (#19771, Stem Cell Technologies). Negatively selected cells contained adherently growing bmMSC, which were expanded for 1–2 weeks in alpha-MEM (#22561-021, Gibco) supplemented with 10% FCS and 1% antibiotic/antimycotic solution until cell differentiation experiments were initiated. Muscle fibroblasts were obtained by outgrowing from rat abdominal muscle and cultured in DMEM supplemented with 10% FCS and 1% antibiotic/antimycotic solution for at least 2 weeks before usage. Human bmMSC were purchased from Life Technologies (#A15652, Thermo Scientific, Waltham, MA, USA) and expanded on collagen type 1-coated plastic dishes by using expansion medium for hMSC (#12746-012, Life Technologies/Thermo Scientific).

### Cell differentiation experiments

The differentiation of isolated HSC, HSC clones, bmMSC and UCBSC was induced by either 2 μM of different bile acids (Sigma-Aldrich, Taufkirchen, Germany) or by 50 ng/ml human FGF4 (#100-31, PreproTech, Rocky Hill, NJ, USA) and 40 ng/ml human HGF (#100-39, PreproTech) dissolved in Iscove’s Modified Dulbecco’s Medium (#12440-046, Gibco) supplemented with 1% insulin-transferrin-sodium selenite (ITS, #I1884, Sigma-Aldrich), 1% linolic acid-albumin from bovine serum (#L9530, Sigma-Aldrich) and 1% antibiotic/antimycotic solution. The differentiation medium was completely exchanged every second to third day. Cells of control experiments were cultured in the same medium, but without bile acids or growth factors. Cell differentiation was monitored in weekly intervals.

### Animal experiments

The two largest liver lobes of female Wistar rats (approximately 250 g body weight) were surgically removed (70% of the liver) after Ketamin/Xylacin narcosis (100 mg/5 mg/kg body weight; Ketavet, PZN-3151811, Zoetis Deutschland GmbH, Berlin, Germany; Rompun, PZN-1320422, Bayer Vital GmbH, Leverkusen, Germany) essentially as described^41^. The blood serum was collected from the portal vein of rats without (0 days) and 2, 4 and 6 days after PHX and stored at −80 °C. The animal experiments were approved by the relevant federal state authority for animal protection (Landesamt für Natur, Umwelt und Verbraucherschutz Nordrhein-Westfalen, Recklinghausen, Germany; reference numbers 9.93.2.10.34.07.163; 50.05-240-27/06) and all animals received care according to the German animal welfare act.

### Cell analysis

For immunofluorescence staining, cultured cells were fixed with either ice-cold methanol for 5 minutes or 4% formalin for 10–20 minutes and incubated with antibodies against keratin 18 (K18, #BM2275P, Acris), keratin 19 (K19, #NB100-687, Novus Biologicals, Littleton, CO, USA; #61029, Progen, Heidelberg, Germany), desmin (#ab8592, Abcam, Cambridge, UK), vimentin (#M0725, Dako, Glostrup, Danmark), Tgr5 (#ab72608, Abcam), Fxr (#417200; Invitrogen), CD44 (#5640S, Cell Signaling, Danvers, MA, USA), CD146 (#2505-1, Epitomics, Burlingame, CA, USA), Hnf4α (#3113S, Cell Signaling) and PDGFRβ (#MA5-15143, Thermo Scientific). Antibodies against Ntcp, Bsep and Oatp4 were kindly provided by Dr. B. Stieger and Dr. P. Meier from the University Hospital Zürich (Switzerland). Appropriate secondary cyanine dye 3 (Cy3) or FITC labelled antibodies (#AP182C, #AP162C, #AP192F, #AP182F, Millipore, Billerica, MA, USA) and 4’,6-diamidino-2-phenylindole (DAPI, #P36935, ProLonged Gold; Molecular Probes, Thermo Scientific) were applied to mark primary antibodies and cell nuclei, respectively. For quantitative reverse transcriptase polymerase chain reaction (qPCR), the first strand cDNA was made from 1 μg total RNA per 20 μl reaction volume using the RevertAid H Minus First Strand cDNA Synthesis Kit (#K1632, Thermo Scientific). The SensiMix SYBR No-ROX Kit (#QT650-05, Bioline, Luckenwalde, Germany) was used for qPCR according to the manufacturer’s instructions. For the amplification of PCR products, 12.5 ng cDNA and 0.6 μmol/l primers (supplemental Table S12, S13) were taken for each reaction. After an initial denaturation at 95 °C, the annealing was carried out at 58 °C and the elongation was performed at 72 °C using 40 cycles (TOptical cycler, Analytic Jena AG, Jena, Germany). All samples were measured in triplicates and hypoxanthine-guanine phosphoribosyltransferase 1 (Hprt1) was used as a reference for the normalization of the results obtained by the 2(−∆∆Ct) method. Albumin was quantified in the culture supernatant by enzyme-linked immunosorbent assays specific for rat, mouse or human albumin (ELISA, #E110-125, #E90-134, #E80-129, Bethyl Laboratories, Montgomery, TX, USA) according to manufacturer’s recommendations. Western blot analysis of protein lysates was performed by semidry blotting according to standard protocols. To obtain nuclear protein fractions, the CNM Compartment Protein Isolation Kit (#3013010, BioCat, Heidelberg, Germany) was used according to the manufacturer’s recommendations. The primary antibodies against Fxr (#417200; Invitrogen), Tgr5 (#ab72608; Abcam) and γ-tubulin (#T5326; Sigma-Aldrich) as well as appropriate secondary antibodies coupled with horseradish peroxidase (#AP182P, #AP192P, Millipore) were used to label protein bands.

### Bile acid analysis

Bile acids of culture supernatants were analysed by UHPLC-MS/MS essentially as described^42^. The system consisted of an UPLC-H class (Waters GmbH, Eschborn, Germany) coupled to a Xevo TQ-S triple quadruple mass spectrometer (Waters GmbH). Electrospray ionization was performed in the negative ionization mode. Chromatographic separation was performed on a BEH column (2.1 mm × 100 mm, 1.7 μm; Waters GmbH). The mobile phase consisted of water containing 0.1% formic acid and 5 mM ammonium acetate (Eluent A) and Acetonitrile (Eluent B). Analytes were separated by a gradient elution. The injection volume was 5 μL and the column was maintained at 40 °C. Detection of the bile acids and their glycine and taurine conjugates was performed in selected reaction monitoring (SRM) mode. Cholic acid (CA), hyocholic acid (HCA), chenodeoxycholic acid (CDCA), deoxycholic acid (DCA), 7-oxo-deoxycholic acid (7-oxo-DCA), murideoxycholic acid (MDCA), lithocholic acid (LCA), lithocholic acid–sulfate (LCA-S), ursodeoxycholic acid (UDCA), hyodeoxycholic acid (HDCA), muricholic acids (α MCA, β MCA, ω MCA), glycocholic acid (GCA), glyohyocholic acid (GHCA), glycochenodeoxycholic acid (GCDCA), glycodeoxycholic acid (GDCA), glycolithocholic acid (GLCA), glycoursodeoxycholic acid (GUDCA), glycohyodeoxycholic acid (GHDCA), taurocholic acid (TCA), taurohyocholic acid (THCA), taurochenodeoxycholic acid (TCDCA), taurodeoxycholic acid (TDCA), taurolithocholic acid (TLCA), tauroursodeoxycholic acid (TUDCA) and taurohyodeoxycholic acid (THDCA) as well as the deuterated BA internal standards (IS) substance d4-CA, d4-GCA and d4-GCDCA were purchased from Sigma-Aldrich and Steraloids (Newport, RI, USA). The taurine conjugated ω MCA, α MCA and β MCA (Steraloids) were confirmed by standards but not separated on UPLC column and hence regarded as T-MCA. The standards for glycine conjugated ω MCA, α MCA, and β MCA were commercially not available and postulated from m/z, retention time and fragmentation as G-MCA.

### Statistics

The significance of the data obtained by rat albumin ELISA and qPCR was determined by analysis of variance (ANOVA) and considered significant at p < 0.05. Significantly different groups are indicated by diverse letters in the graphs, whereas non-significant groups share similar letters. Single values, which differ significantly from all others, are indicated by asterisks. The results of at least 3 independent experiments were expressed as mean values in percent or as concentrations. The variance was specified as standard error of mean (±SEM).

## Additional Information

**How to cite this article**: Sawitza, I. *et al.* Bile acids induce hepatic differentiation of mesenchymal stem cells. *Sci. Rep.* **5**, 13320; doi: 10.1038/srep13320 (2015).

## Supplementary Material

Supplementary Information
