# Supplementary material for: Bile acids induce hepatic differentiation of mesenchymal stem cells
Source: Sci Rep. 2015 Aug 25;5:13320. doi: 10.1038/srep13320 (PMC4548444; doi:10.1038/srep13320)
Supplement: Supplementary Information [file srep13320-s1.doc]

**Supplemental Tables and Figures**

**Bile acids induce hepatic differentiation of mesenchymal stem cells**

**Authors**

Iris Sawitza ^a^, Claus Kordes ^a^, Silke Götze ^a^, Diran Herebian ^b^ and Dieter Häussinger ^a *^

**Author affiliations**

^a^Clinic of Gastroenterology, Hepatology and Infectious Diseases,

^b^Department of General Pediatrics, Neonatology and Pediatric Cardiology,

Heinrich Heine University, Moorenstraße 5, 40225 Düsseldorf, Germany

**^*^Corresponding author**

Prof. Dr. D. Häussinger, Clinic of Gastroenterology, Hepatology and Infectious Diseases, Heinrich Heine University, Moorenstraße 5, 40225 Düsseldorf, Germany

e-mail: haeussin@uni-duesseldorf.de; phone: 00492118117569; fax: 00492118118752

**Supplemental Table S1|** **Bile acid concentrations in the blood serum of rats during the first 6 days after PHX.** The SEM is indicated in brackets (n = 3).

| **[nmol/L]** | **control** | **2 days** | **4 days** | **6 days** |
| --- | --- | --- | --- | --- |
| **CA** | 3598 (± 284) | 23511 (± 615) | 16849 (± 1237) | 15145 (± 606) |
| **CDCA** | 389 (± 46) | 3210 (± 167) | 3173 (± 265) | 2130 (± 182) |
| **DCA** | 271 (± 20) | 5933 (± 516) | 1929 (± 112) | 1585 (± 70) |
| **UDCA** | 157 (± 15) | 324 (± 18) | 444 (± 49) | 501 (± 61) |
| **HDCA** | 54 (± 11) | 1022 (± 82) | 712 (± 86) | 523 (± 9) |
| **α/ω-MCA** | 696 (± 33) | 7590 (± 663) | 4997 (± 393) | 3837 (± 380) |
| **β-MCA** | 3030 (± 125) | 34859 (± 1780) | 5464 (± 390) | 7505 (± 478) |
| **LCA** | 26 (± 1) | 56 (± 4) | 71 (± 5) | 53 (± 4) |
| **LCAS** | 8 (± 1) | 13 (± 0) | 10 (± 1) | 11 (± 0) |
| **GCA** | 8756 (± 196) | 15831 (± 623) | 6695 (± 25) | 5794 (± 314) |
| **GMCA** | 3519 (± 71) | 2920 (± 61) | 2413 (± 37) | 1691 (± 43) |
| **GUDCA** | 184 (± 15) | 34 (± 6) | 36 (± 14) | 120 (± 8) |
| **GHDCA** | 8 (± 1) | 95 (± 6) | 99 (± 5) | 84 (± 1) |
| **GCDCA** | 425 (± 29) | 1307 (± 134) | 1061 (± 75) | 811 (± 27) |
| **GDCA** | 83 (± 5) | 1709 (± 149) | 632 (± 44) | 393 (± 29) |
| **GLCA** | 3 (± 1) | 5 (± 1) | 7 (± 3) | 5 (± 1) |
| **TCA** | 15142 (± 765) | 32392 (± 2645) | 23997 (± 948) | 16271 (± 911) |
| **TMCA** | 2540 (± 167) | 8992 (± 270) | 10795 (± 471) | 2083 (± 240) |
| **TUDCA** | 506 (± 11) | 848 (± 14) | 905 (± 31) | 330 (± 36) |
| **THDCA** | 55 (± 2) | 150 (± 5) | 566 (± 45) | 161 (± 30) |
| **TCDCA** | 1523 (± 70) | 3346 (± 303) | 721 (± 70) | 2073 (± 88) |
| **TDCA** | 1487 (± 164) | 1533 (± 57) | 1327 (± 34) | 591 (± 56) |
| **TLCA** | 25 (± 3) | 13 (± 0) | 16 (± 1) | 17 (± 1) |

**Supplemental Table S2|** **Bile acid concentrations in the blood serum of rats during the first 6 days after PHX.** The SEM is indicated in brackets (n = 3). The values were compared to untreated control rats, which were set to 100%.

| **[%]** | **control** | **2 days** | **4 days** | **6 days** |
| --- | --- | --- | --- | --- |
| **CA** | 100 (± 8) | 653 (± 17) | 468 (± 34) | 421 (± 17) |
| **CDCA** | 100 (± 12) | 824 (± 43) | 815 (± 68) | 547 (± 47) |
| **DCA** | 100 (± 7) | 2190 (± 190) | 712 (± 41) | 585 (± 26) |
| **UDCA** | 100 (± 9) | 206 (± 11) | 282 (± 31) | 319 (± 39) |
| **HDCA** | 100 (± 20) | 1878 (± 150) | 1308 (± 158) | 962 (± 16) |
| **α/ω-MCA** | 100 (± 5) | 1090 (± 95) | 718 (± 56) | 551 (± 55) |
| **β-MCA** | 100 (± 4) | 1151 (± 59) | 180 (± 13) | 248 (± 16) |
| **LCA** | 100 (± 5) | 213 (± 16) | 268 (± 18) | 202 (± 15) |
| **LCAS** | 100 (± 9) | 158 (± 6) | 127 (± 9) | 142 (± 6) |
| **GCA** | 100 (± 2) | 181 (± 7) | 76 (± 0) | 66 (± 4) |
| **GMCA** | 100 (± 2) | 83 (± 2) | 69 (± 1) | 48 (± 1) |
| **GUDCA** | 100 (± 8) | 18 (± 3) | 20 (± 8) | 65 (± 4) |
| **GHDCA** | 100 (± 9) | 1253 (± 72) | 1308 (± 66) | 1102 (± 10) |
| **GCDCA** | 100 (± 7) | 307 (± 32) | 249 (± 18) | 191 (± 6) |
| **GDCA** | 100 (± 6) | 2066 (± 179) | 764 (± 53) | 474 (± 35) |
| **GLCA** | 100 (± 20) | 139 (± 39) | 203 (± 78) | 135 (± 39) |
| **TCA** | 100 (± 5) | 214 (± 17) | 158 (± 6) | 107 (± 6) |
| **TMCA** | 100 (± 7) | 354 (± 11) | 425 (± 19) | 82 (± 9) |
| **TUDCA** | 100 (± 6) | 167 (± 7) | 179 (± 6) | 65 (± 7) |
| **THDCA** | 100 (± 3) | 270 (± 9) | 1019 (± 80) | 290 (± 53) |
| **TCDCA** | 100 (± 5) | 220 (± 20) | 47 (± 5) | 136 (± 6) |
| **TDCA** | 100 (± 11) | 103 (± 4) | 89 (± 2) | 40 (± 4) |
| **TLCA** | 100 (± 13) | 51 (± 1) | 64 (± 3) | 67 (± 5) |

**Supplemental Table S3| Primary cultured HSC, clonally expanded HSC and UCBSC, but not muscle fibroblasts differentiate into hepatocyte-like cells in response to TUDCA treatment. Primary cultures of HSC, HSC clones (5G4, 4E7, 2G8), UCBSC clone (1G11) and muscle fibroblasts from rats were treated with serum-free culture medium without (control) or with 2 µM TUDCA for 21 days. The expression of the hepatocyte marker albumin was determined by qPCR in cell lysates from three independent experiments. The obtained values were normalized for Hprt1 mRNA in the samples. Data are expressed as percentage of the maximal relative expression found in cultured hepatocytes (100 ± 8.3%; n = 3).**

| **albumin mRNA [% ± SEM]** | | | | | | | | |
| --- | --- | --- | --- | --- | --- | --- | --- | --- |
| **culture time** | **HSC** | | **HSC clones** | | **UCBSC clone** | | **muscle fibroblasts** | |
|  | **control** | **TUDCA** | **control** | **TUDCA** | **control** | **TUDCA** | **control** | **TUDCA** |
| **1 day** | 0.008  (± 0.002) | 0.008  (± 0.002) | 0.003  (± 0.0007) | 0.003  (± 0.0002) | 0.001  (± 0.0003) | 0.001  (± 0.0003) | 0.002  (± 0.0007) | 0.002  (± 0.0007) |
| **7 days** | 0.006  (± 0.003) | 5.9  (± 0.5) | 0.002  (± 0.0002) | 5.7  (± 0.6) | 0.002  (± 0.0003) | 1.9  (± 0.1) | 0.002  (± 0.0006) | 0.003  (± 0.0009) |
| **14 days** | 0.007  (± 0.002) | 14.8  (± 0.9) | 0.002  (± 0.0005) | 8.8  (± 0.8) | 0.001  (± 0.0002) | 4.0  (± 0.3) | 0.003  (± 0.0002) | 0.003  (± 0.0009) |
| **21 days** | 0.005  (± 0.001) | 22.8  (± 2.3) | 0.002  (± 0.0004) | 12.3  (± 0.7) | 0.001  (± 0.0003) | 6.5  (± 0.6) | 0.003  (± 0.0006) | 0.003  (± 0.0006) |

**Supplemental Table S4| TUDCA-triggered hepatic differentiation of HSC is supported by Wnt3a.** Primary cultures of HSC from rats were treated with serum-free culture medium without (control) or with 2 µM TUDCA for 21 days. Wnt3a (10 ng/ml) was added alone or in combination with 2 µM TUDCA. The expression of the hepatocyte marker albumin was determined by qPCR in cell lysates of three independent experiments. The obtained values were normalized for Hprt1 mRNA in the samples. Data are expressed as percentage of the maximal relative expression found in cultured hepatocytes (100 ± 9.9%; n = 3).

| **albumin mRNA [% ± SEM]** | | | | |
| --- | --- | --- | --- | --- |
| **culture time** | **HSC** | | | |
|  | **control** | **TUDCA** | **Wnt3a** | **TUDCA+ Wnt3a** |
| **1 day** | 0.01  (± 0.003) | 0.01  (± 0.003) | 0.01  (± 0.003) | 0.01  (± 0.003) |
| **7 days** | 0.01  (± 0.001) | 10.3  (± 1.2) | 2.5  (± 0.4) | 31.5  (± 2.3) |
| **14 days** | 0.01  (± 0.003) | 16.3  (± 1.4) | 4.8  (± 0.5) | 43.2  (± 2.8) |
| **21 days** | 0.01  (± 0.002) | 20.5  (± 1.5) | 7.2  (± 0.7) | 58.3  (± 5.2) |

**Supplemental Table S5| Effect of TUDCA (2 µM) on albumin production by primary cultured HSC, clonally expanded HSC and UCBSC. Primary cultures of HSC, HSC clones (5G4, 4E7, 2G8), UCBSC clone (1G11) and muscle fibroblasts from rats were treated with serum-free culture medium without (control) or with 2 µM TUDCA for 21 days. The protein levels of the hepatocyte marker albumin was determined in the culture supernatants by a rat albumin-specific ELISA (n = 3*; n = 9**).**

| **albumin protein [mg/ml ± SEM]** | | | | | | | | |
| --- | --- | --- | --- | --- | --- | --- | --- | --- |
| **culture time** | **HSC**** | | **HSC clones*** | | **UCBSC clone*** | | **muscle fibroblasts*** | |
|  | **control** | **TUDCA** | **control** | **TUDCA** | **control** | **TUDCA** | **control** | **TUDCA** |
| **1 day** | 0  (± 0) | 0  (± 0) | 0  (± 0) | 0  (± 0) | 0  (± 0) | 0  (± 0) | 0  (± 0) | 0  (± 0) |
| **7 days** | 0  (± 0) | 0.5  (± 0.03) | 0  (± 0) | 0.2  (± 0.03) | 0  (± 0) | 0.2  (± 0.03) | 0  (± 0) | 0  (± 0) |
| **14 days** | 0  (± 0) | 0.9  (± 0.09) | 0  (± 0) | 0.4  (± 0.02) | 0  (± 0) | 0.3  (± 0.01) | 0  (± 0) | 0  (± 0) |
| **21 days** | 0  (± 0) | 1.5  (± 0.09) | 0  (± 0) | 0.8  (± 0.2) | 0  (± 0) | 0.6  (± 0.1) | 0  (± 0) | 0  (± 0) |

**Supplemental Table S6| Primary cultured HSC, clonally expanded HSC and UCBSC, but not muscle fibroblasts differentiate into hepatocyte-like cells in response to TUDCA treatment. Primary cultures of HSC, HSC clones (5G4, 4E7, 2G8), UCBSC clone (1G11) and muscle fibroblasts from rats were treated with serum-free culture medium without (control) or with 2 µM TUDCA for 21 days. The expression of the hepatocyte marker Hnf4α was determined by qPCR in cell lysates of three independent experiments. The obtained values were normalized for Hprt1 mRNA in the samples. Data are expressed as percentage of the maximal relative expression found in cultured hepatocytes (100 ± 7.4%; n = 3).**

| **HNF4α mRNA [% ± SEM]** | | | | | | | | |
| --- | --- | --- | --- | --- | --- | --- | --- | --- |
| **culture time** | **HSC** | | **HSC clones** | | **UCBSC clone** | | **muscle fibroblasts** | |
|  | **control** | **TUDCA** | **control** | **TUDCA** | **control** | **TUDCA** | **control** | **TUDCA** |
| **1 day** | 0.002  (± 0.0004) | 0.002  (± 0.0004) | 0.0009  (± 0.0002) | 0.0009  (± 0.0002) | 0.0006  (± 0.0002) | 0.0006  (± 0.0002) | 0.0002  (± 0.00004) | 0.0002  (± 0.00004) |
| **7 days** | 0.002  (± 0.0005) | 2.2  (± 0.2) | 0.001  (± 0.0002) | 1.5  (± 0.3) | 0.0004  (± 0.0001) | 1.0  (± 0.09) | 0.0008  (± 0.00009) | 0.001  (± 0.0003) |
| **14 days** | 0.002  (± 0.0009) | 4.3  (± 0.4) | 0.0006  (± 0.0001) | 3.1  (± 0.4) | 0.0004  (± 0.0001) | 1.6  (± 0.3) | 0.0009  (± 0.0002) | 0.0008  (± 0.0002) |
| **21 days** | 0.002  (± 0.0009) | 9.5  (± 0.9) | 0.0007  (± 0.0003) | 3.9  (± 0.5) | 0.0003  (± 0.0001) | 2.4  (± 0.4) | 0.0009  (± 0.0003) | 0.0008  (± 0.0002) |

**Supplemental Table S7| Primary cultured HSC, clonally expanded HSC and UCBSC, but not muscle fibroblasts differentiate into hepatocyte-like cells in response to TUDCA treatment. Primary cultures of HSC, HSC clones (5G4, 4E7, 2G8), UCBSC clone (1G11) and muscle fibroblasts from rats were treated with serum-free culture medium without (control) or with 2 µM TUDCA for 21 days. The expression of the hepatocyte marker Cyp7a1 was determined by qPCR in cell lysates of three independent experiments. The obtained values were normalized for Hprt1 mRNA in the samples. Data are expressed as percentage of the maximal relative expression found in cultured hepatocytes (100 ± 9.2%; n = 3).**

| **Cyp7a1 mRNA [% ± SEM]** | | | | | | | | |
| --- | --- | --- | --- | --- | --- | --- | --- | --- |
| **culture time** | **HSC** | | **HSC clones** | | **UCBSC clone** | | **muscle fibroblasts** | |
|  | **control** | **TUDCA** | **control** | **TUDCA** | **control** | **TUDCA** | **control** | **TUDCA** |
| **1 day** | 0.01  (± 0.0003) | 0.01  (± 0.0003) | 0.003  (± 0.0003) | 0.003  (± 0.0003) | 0.0007  (± 0.0002) | 0.0007  (± 0.0002) | 0.003  (± 0.0007) | 0.003  (± 0.0007) |
| **7 days** | 0.01  (± 0.0006) | 1.9  (± 0.04) | 0.004  (± 0.0008) | 2.3  (± 0.1) | 0.0008  (± 0.0001) | 0.9  (± 0.1) | 0.002  (± 0.0005) | 0.003  (± 0.001) |
| **14 days** | 0.01  (± 0.001) | 7.9  (± 0.4) | 0.004  (± 0.0006) | 5.1  (± 0.4) | 0.0006  (± 0.0001) | 1.7  (± 0.3) | 0.003  (± 0.0009) | 0.003  (± 0.0008) |
| **21 days** | 0.004  (± 0.0009) | 12.0  (± 0.7) | 0.004  (± 0.0009) | 8.6  (± 0.6) | 0.0006  (± 0.0003) | 2.6  (± 0.3) | 0.003  (± 0.0009) | 0.003  (± 0.0009) |

**Supplemental Table S8| Primary cultured HSC, clonally expanded HSC and UCBSC, but not muscle fibroblasts differentiate into hepatocyte-like cells in response to TUDCA treatment. Primary cultures of HSC, HSC clones (5G4, 4E7, 2G8), UCBSC clone (1G11) and muscle fibroblasts from rats were treated with serum-free culture medium without (control) or with 2 µM TUDCA for 21 days. The expression of the stellate cell/MSC marker desmin was determined by qPCR in cell lysates of three independent experiments. The obtained values were normalized for Hprt1 mRNA in the samples. Data are expressed as percentage of the maximal relative expression found in primary HSC (100 ± 5.9%; n = 3).**

| **desmin mRNA [% ± SEM]** | | | | | | | | |
| --- | --- | --- | --- | --- | --- | --- | --- | --- |
| **culture time** | **HSC** | | **HSC clones** | | **UCBSC clone** | | **muscle fibroblasts** | |
|  | **control** | **TUDCA** | **control** | **TUDCA** | **control** | **TUDCA** | **control** | **TUDCA** |
| **1 day** | 100  (± 5.9) | 100.0  (± 5.9) | 10.5  (± 0.7) | 10.5  (± 0.7) | 6.4  (± 0.4) | 6.4  (± 0.4) | 0.001  (± 0.0006) | 0.001  (± 0.0006) |
| **7 days** | 111.1  (± 7.6) | 88.7  (± 4.2) | 10.5  (± 0.5) | 9.2  (± 0.6) | 6.5  (± 0.4) | 5.4  (± 0.3) | 0.002  (± 0.0006) | 0.001  (± 0.0003) |
| **14 days** | 127.7  (± 5.0) | 69.6  (± 5.0) | 10.9  (± 0.6) | 8.3  (± 0.7) | 6.4  (± 0.4) | 4.3  (± 0.3) | 0.002  (± 0.0005) | 0.001  (± 0.0007) |
| **21 days** | 151.4  (± 6.2) | 51  (± 4.0) | 10.5  (± 0.4) | 6.6  (± 0.5) | 6.5  (± 0.4) | 3.8  (± 0.3) | 0.001  (± 0.0004) | 0.002  (± 0.0006) |

**Supplemental Table S9| Primary cultured HSC, clonally expanded HSC and UCBSC, but not muscle fibroblasts differentiate into hepatocyte-like cells in response to TUDCA treatment. Primary cultures of HSC, HSC clones (5G4, 4E7, 2G8), UCBSC clone (1G11) and muscle fibroblasts from rats were treated with serum-free culture medium without (control) and with 2 µM TUDCA for 21 days. The expression of the progenitor cell and bile duct cell marker K19 was determined by qPCR in cell lysates of three independent experiments. The obtained values were normalized for Hprt1 mRNA in the samples. Data are expressed as percentage of the maximal relative expression found in primary HSC (100 ± 7.7%; n = 3).**

| **K19 mRNA [% ± SEM]** | | | | | | | | |
| --- | --- | --- | --- | --- | --- | --- | --- | --- |
| **culture time** | **HSC** | | **HSC clones** | | **UCBSC clone** | | **muscle fibroblasts** | |
|  | **control** | **TUDCA** | **control** | **TUDCA** | **control** | **TUDCA** | **control** | **TUDCA** |
| **1 day** | 0.05  (± 0.005) | 0.05  (± 0.005) | 0.02  (± 0.003) | 0.02  (± 0.003) | 0.003  (± 0.001) | 0.003  (± 0.001) | 0.03  (± 0.01) | 0.03  (± 0.01) |
| **7 days** | 0.06  (± 0.008) | 41.3  (± 3.1) | 0.02  (± 0.004) | 44.2  (± 3.9) | 0.002  (± 0.0005) | 23.4  (± 2.8) | 0.02  (± 0.009) | 0.02  (± 0.008) |
| **14 days** | 0.2  (± 0.006) | 100.0  (± 7.7) | 0.02  (± 0.002) | 81.4  (± 5.3) | 0.002  (± 0.0001) | 52.8  (± 3.6) | 0.002  (± 0.0001) | 0.03  (± 0.01) |
| **21 days** | 0.09  (± 0.005) | 75.4  (± 6.4) | 0.03  (± 0.005) | 57.2  (± 4.4) | 0.002  (± 0.0006) | 34.8  (± 2.2) | 0.03  (± 0.007) | 0.03  (± 0.007) |

**Supplemental Table S10| Primary cultured HSC, clonally expanded HSC and UCBSC, but not muscle fibroblasts differentiate into hepatocyte-like cells in response to TUDCA treatment. Primary cultures of HSC, HSC clones (5G4, 4E7, 2G8), UCBSC clone (1G11) and muscle fibroblasts from rats were treated with serum-free culture medium without (control) and with 2 µM TUDCA for 21 days. The expression of the progenitor cell and bile duct cell marker Epcam was determined by qPCR in cell lysates of three independent experiments. The obtained values were normalized for Hprt1 mRNA in the samples. Data are expressed as percentage of the maximal relative expression found in primary HSC (100 ± 7.6%; n = 3).**

| **Epcam mRNA [% ± SEM]** | | | | | | | | |
| --- | --- | --- | --- | --- | --- | --- | --- | --- |
| **culture time** | **HSC** | | **HSC clones** | | **UCBSC clone** | | **muscle fibroblasts** | |
|  | **control** | **TUDCA** | **control** | **TUDCA** | **control** | **TUDCA** | **control** | **TUDCA** |
| **1 day** | 0.05  (± 0.003) | 0.05  (± 0.003) | 0.009  (± 0.001) | 0.009  (± 0.001) | 0.002  (± 0.0005) | 0.002  (± 0.0005) | 0.02  (± 0.007) | 0.02  (± 0.007) |
| **7 days** | 0.1  (± 0.008) | 35.4  (± 1.9) | 0.01  (± 0.001) | 35.1  (± 2.1) | 0.003  (± 0.0002) | 31.7  (± 3.7) | 0.02  (± 0.008) | 0.04  (± 0.02) |
| **14 days** | 0.1  (± 0.02) | 100.0  (± 7.6) | 0.02  (± 0.002) | 83.6  (± 4.9) | 0.003  (± 0.0003) | 55.6  (± 2.9) | 0.04  (± 0.007) | 0.04  (± 0.01) |
| **21 days** | 0.4  (± 0.03) | 62.7  (± 4.3) | 0.02  (± 0.002) | 49.3  (± 5.1) | 0.002  (± 0.0004) | 42.6  (± 3.4) | 0.04  (± 0.008) | 0.03  (± 0.01) |

**Supplemental Table S11| Primary cultured HSC, clonally expanded HSC and UCBSC, but not muscle fibroblasts differentiate into hepatocyte-like cells in response to TUDCA treatment. Primary cultures of HSC, HSC clones (5G4, 4E7, 2G8), UCBSC clone (1G11) and muscle fibroblasts from rats were treated with serum-free culture medium without (control) or with 2 µM TUDCA for 21 days. The expression of the progenitor cell marker Afp was determined by qPCR in cell lysates of three independent experiments. The obtained values were normalized for Hprt1 mRNA in the samples. Data are expressed as percentage of the maximal relative expression found in primary HSC (100 ± 3.3%; n = 3)**

| **Afp mRNA [% ± SEM]** | | | | | | | | |
| --- | --- | --- | --- | --- | --- | --- | --- | --- |
| **culture time** | **HSC** | | **HSC clones** | | **UCBSC clone** | | **muscle fibroblasts** | |
|  | **control** | **TUDCA** | **control** | **TUDCA** | **control** | **TUDCA** | **control** | **TUDCA** |
| **1 day** | 0.05  (± 0.007) | 0.05  (± 0.007) | 0.01  (± 0.003) | 0.01  (± 0.003) | 0.005  (± 0.001) | 0.005  (± 0.001) | 0.02  (± 0.002) | 0.02  (± 0.002) |
| **7 days** | 0.01  (± 0.003) | 47.7  (± 2.5) | 0.01  (± 0.003) | 32.5  (± 3.1) | 0.004  (± 0.001) | 21.4  (± 1.3) | 0.02  (± 0.007) | 0.03  (± 0.004) |
| **14 days** | 0.7  (± 0.06) | 100.0  (± 3.3) | 0.02  (± 0.003) | 60.1  (± 3.8) | 0.003  (± 0.002) | 36.4  (± 1.9) | 0.03  (± 0.01) | 0.02  (± 0.007) |
| **21 days** | 0.6  (± 0.07) | 46.7  (± 2.7) | 0.02  (± 0.004) | 41.3  (± 4.2) | 0.002  (± 0.0004) | 27.5  (± 2.7) | 0.02  (± 0.005) | 0.03  (± 0.006) |

**Supplemental Table S12**| Rat primer sets for qPCR

| **Gene** | **Forward Primer** | **Reverse Primer** | **bp** | **Accession No.** |
| --- | --- | --- | --- | --- |
| **α-Sma** | GCACTACCATGTACCCAGGCA | TGCGTTCTGGAGGAGCAATA | 102 | NM_031004 |
| **Afp** | ACCTGACAGGGAAGATGGTG | GCAGTGGTTGATACCGGAGT | 155 | NM_012493 |
| **albumin** | CTTCAAAGCCTGGGCAGTAG | GCACTGGCTTATCACAGCAA | 221 | V01222 |
| **Asbt** | TGGGCTTCCTCTGTCAGTTT | TTCCAAGAGCAAGCAGTGTG | 120 | NM_017222 |
| **axin2** | GAGTGAGCGTCAGAGCAAGTC | GTCCTGGGTAAATGGGTGAG | 169 | NM_024355 |
| **Bsep** | TACCAGGAAAAGCGTGTGTG | CCCAGTGATGACCCATAACC | 197 | NM_031760 |
| **Cyp7a1** | CACCATTCCTGCAACCTTTT | GTACCGGCAGGTCATTCAGT | 170 | NM_012942 |
| **desmin** | AGCCTGGGTCAGAGACAGAA | TATCTCCTGCTCCCACATCC | 166 | NM_022531 |
| **Epcam** | TGCATACTGCACTTCAGGACA | GGAACAAGGACTCCCCCTTTA | 195 | NM_138541 |
| **Fxr** | CTCTGCTCACAGCAATTGTCA | TTGAATGTCCGGAGTTCTGTC | 184 | NM_021745 |
| **Gli1** | CTCCTCGGAGTTCAGTCAAACT | CTAGACATGTCCCCTTCCAAAG | 394 | NM_001191910 |
| **Hes1** | ACCGGACAAACCAAAGACAG | CCAGAATGTCTGCCTTCTCC | 173 | NM_024360 |
| **Hnf4α** | AAATGTGCAGGTGTTGACCA | CACGCTCCTCCTGAAGAATC | 178 | NM_022180 |
| **Hprt1** | AAGTGTTGGATACAGGCCAGA | GGCTTTGTACTTGGCTTTTCC | 145 | NM_012583 |
| **K19** | AGTAACGTGCGTGCTGACAC | AGTCGCACTGGTAGCAAGGT | 193 | NM_199498 |
| **notch3** | CTACCTTGGCTCTGCTGAAAA | AGCCTGTCCAAGTGATCTGTG | 160 | NM_020087 |
| **Oatp4** | TTCTGGTTTGATTGATGGAGGC | ATGAAGCAGCCAATTCCAATCA | 120 | AJ271682 |
| **Smad2** | GTGTGTGTGAACCCTTACCAC | TGGTGGTGTTTCTGGGATGTA | 189 | NM_019191 |
| **Smad5** | AATCGCAACTCAACCATCGAG | TGGAAGCCATGGTGAAAGTTG | 149 | NM_021692 |
| **Tgr5** | TTCTCTCTGTCCGAGTGTTGG | CACAGCAAAAAGAGCAGTGTG | 160 | NM_177936 |
| **vimentin** | GAACGTAAAGTGGAATCCTTGC | GTCTCCGGTATTCGTTTGACTC | 304 | NM_031140 |
| **Wnt5a** | CCTTCGCCCAGGTTGTAATAA | ACTGCATGTGGTCCTGATACA | 171 | NM_022631 |
| **Wnt11** | TGCTTGACCTGGAGAGAGGT | GGAGACCGTAGCTGAGGTTG | 195 | NM_080401 |

**Supplemental Table S13**| Mouse primer sets for qPCR

| **Gene** | **Forward Primer** | **Reverse Primer** | **bp** | **Accession No.** |
| --- | --- | --- | --- | --- |
| **albumin** | AATTGGCAACAGACCTGACC | CCTCAACAAAATCAGCAGCA | 237 | NM_009654 |
| **Fxr** | CTGTACCCTGCCATCCAAGT | TCATGGATTTTTGGTGAGCA | 157 | AY094586 |
| **Hprt1** | CAGCGTTTCTGAGCCATTGCT | ATCCTCGGCATAATGATTAGGT | 226 | NM_013556 |
| **Tgr5** | TGGAAGTTTATGGCCTCCTG | CCAACACAGCAAGAAGAGCA | 209 | NM_174985 |


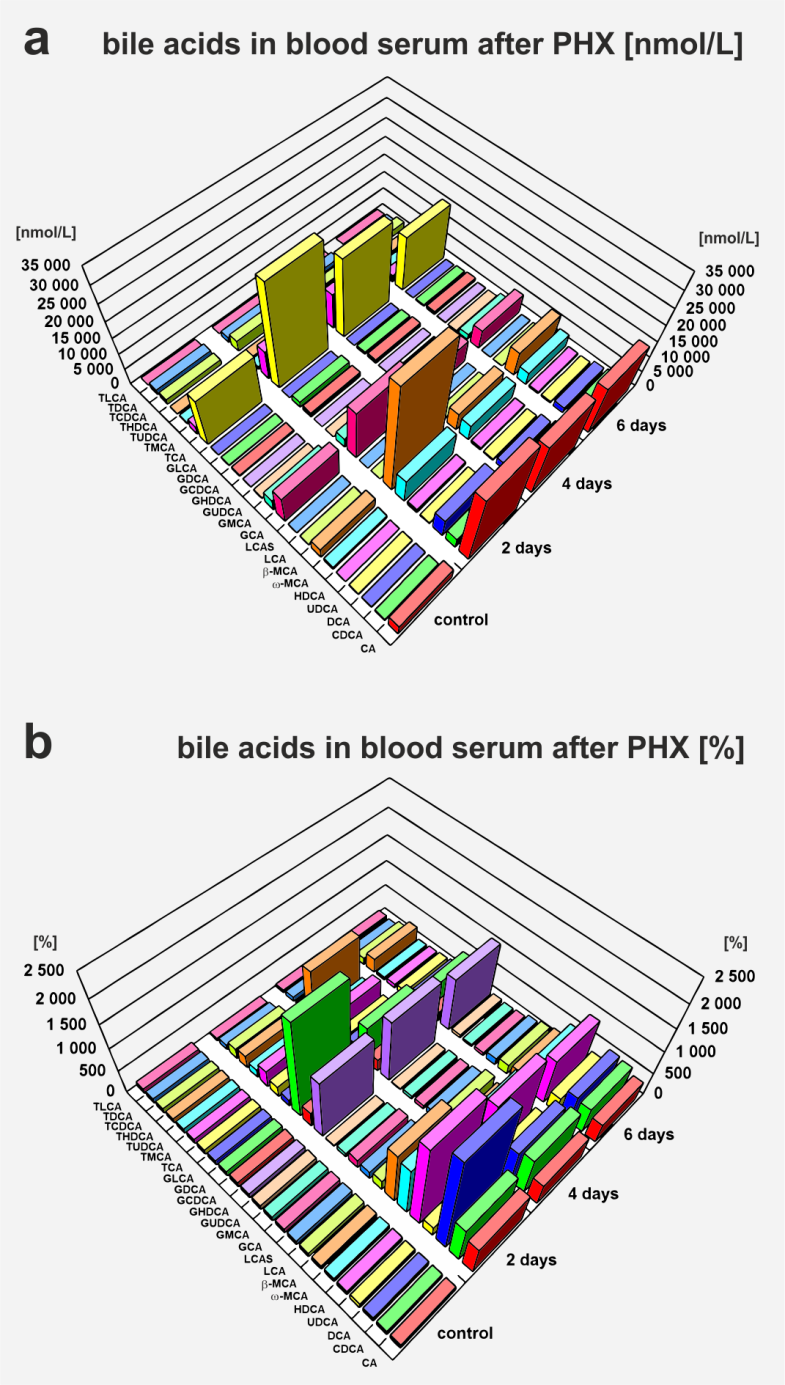


**Supplemental Figure S1| Quantification of bile acids in the blood serum of rats during liver regeneration after PHX.** (**a**) Absolut concentration [nmol/L] and (**b**) relative increase of bile acids in the blood serum of rats that underwent PHX compared to untreated control rats [%]. The bile acids were measured by UHPLC-MS/MS (n = 3).


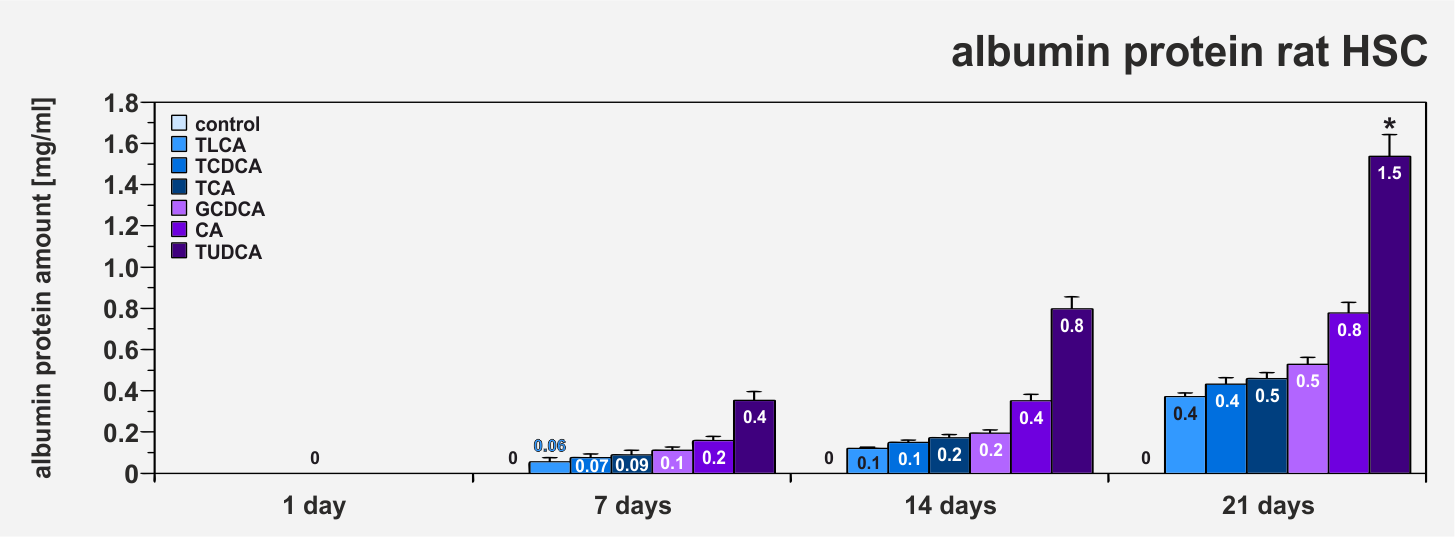


**Supplemental Figure S2| Bile acids promote hepatic differentiation of rat HSC.** Freshly isolated rat HSCs were treated with 2 µM TLCA, TCDCA, TCA, GCDCA, CA or TUDCA for 21 days in serum-free media (n = 3). The hepatocyte marker albumin was quantified in culture supernatants by a rat albumin-specific ELISA. The gradual increase of the hepatocyte marker albumin at the protein level in HSC cultures indicated that stellate cells differentiated into liver parenchymal cells in response to bile acid treatment. Albumin release was not observed in the culture supernatants of control HSC, which received the same medium but without bile acids.


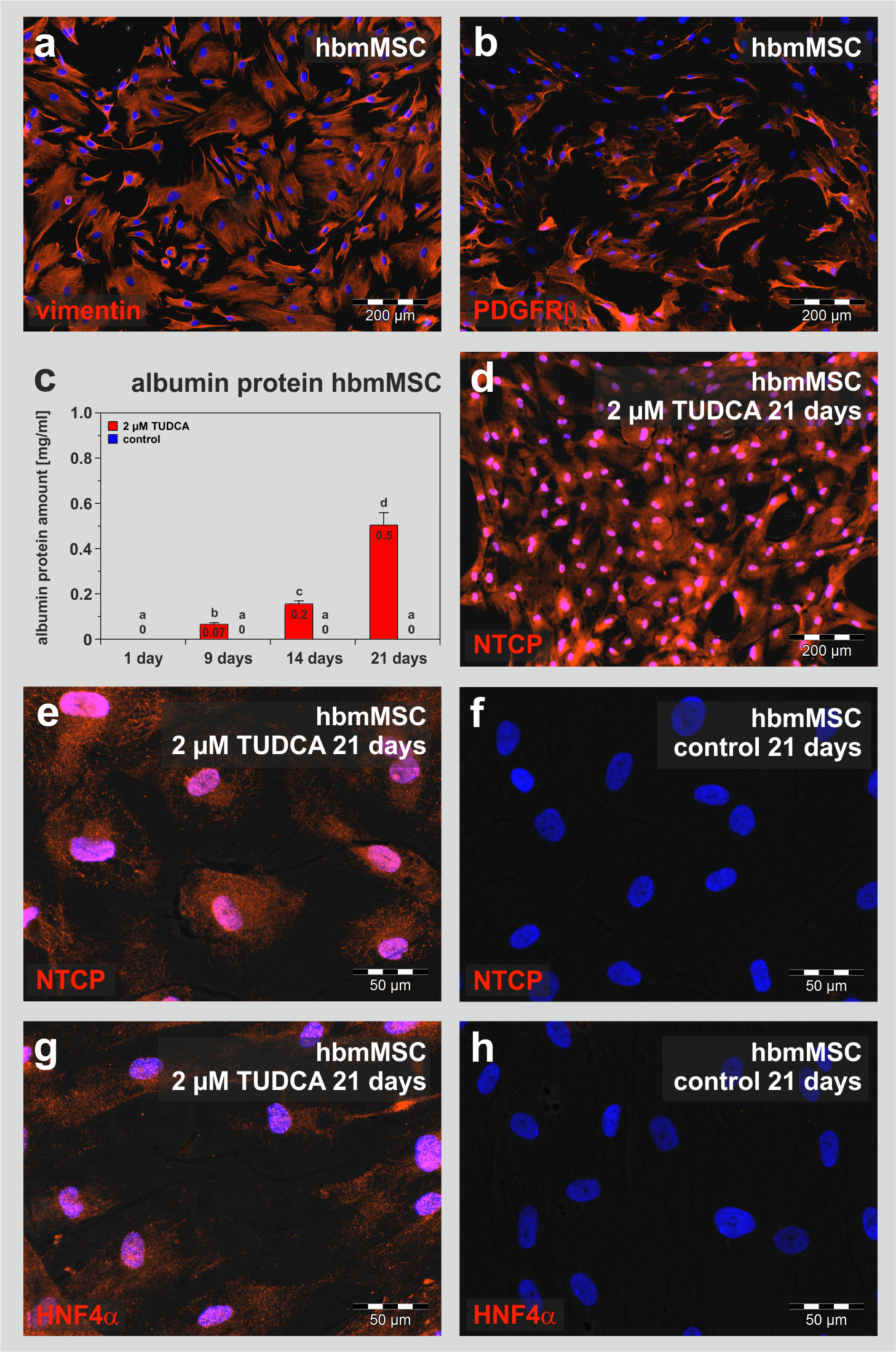


**Supplemental Figure S3|** **TUDCA-mediated hepatic differentiation in MSC from human bone marrow (hbmMSC).** Characterization of isolated hbmMSC by immunofluorescence using antibodies against the MSC marker proteins (**a**) vimentin and (**b**) PDGFRβ (red; passage 6). The hbmMSC were also uniformly positive for CD44 and CD146 (not shown), which supports their classification as MSC. (**c**) The hbmMSC were treated with 2 µM TUDCA for 21 days and the hepatocyte marker albumin was quantified in culture supernatants by an ELISA specific for human albumin. The gradual increase of the protein albumin indicated that hbmMSC differentiated into hepatocyte-like cells in response to bile acid treatment. Albumin release was not observed in the culture supernatants of control hbmMSC, which received the same medium but without bile acids. (**d, e**) TUDCA-mediated hepatic differentiation was found in a large proportion of hbmMSC as indicated by immunofluorescence of the hepatocyte marker NTCP (red), (**f**) whereas hbmMSC of the control lacked NTCP expression after 21 days of culture. (**g**) Also the hepatocyte marker HNF4α was found in the nuclei of hbmMSC treated with TUDCA for 21 days (red). (**f**) Without TUDCA treatment the hbmMSC remained negative for HNF4α immunofluorescence.


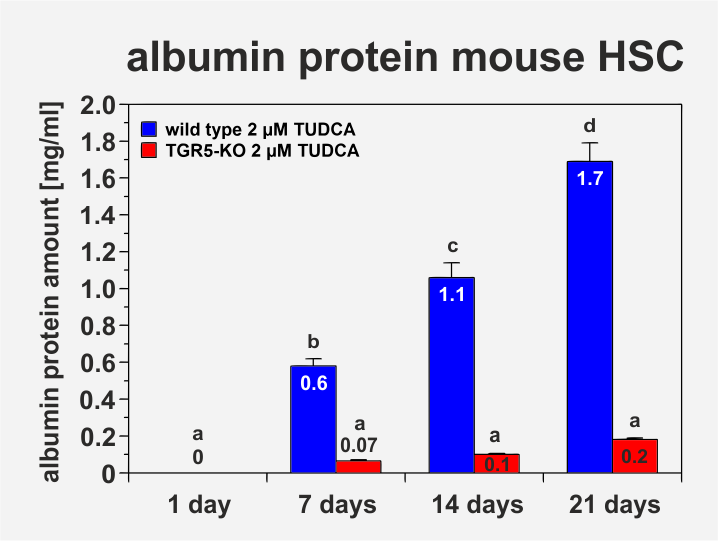


**Supplemental Figure S4|** **Tgr5 is required for TUDCA-mediated hepatic differentiation of mouse HSC.** Isolated HSC from wild type and Tgr5-KO mice were treated with 2 µM TUDCA for 21 days (n = 4-5). The release of albumin was measured in culture supernatants by an ELISA specific for mouse albumin. A significant induction and up-regulation of albumin by TUDCA treatment was only observed in wild type HSC. Stellate cells from wild type and Tgr5-KO mice treated with the same medium but without TUDCA showed no albumin release (not shown).


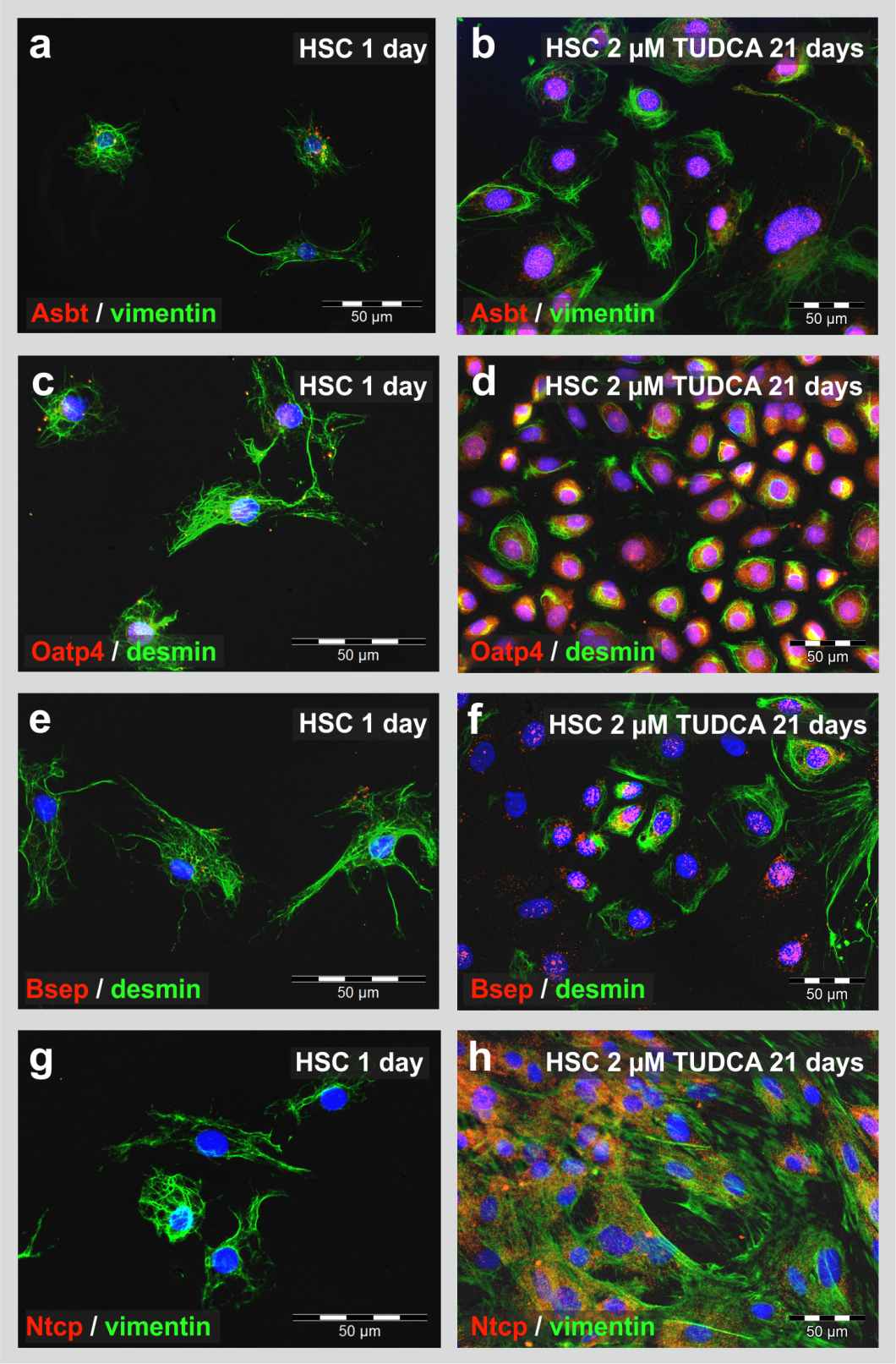


**Supplemental Figure S5| Immunofluorescence staining of bile acid transporters in freshly isolated and TUDCA-treated HSC from rats.** The presence of bile acid transporters in (**a, c, e, g**) freshly isolated and (**b, d, f, h**) TUDCA-treated HSC was investigated by immunofluorescence staining. Freshly isolated HSC from rats were treated with 2 µM TUDCA for 21 days. Asbt (red) was detectable at protein level in HSC (**a**) shortly after isolation (**b**) and TUDCA treatment. Only weak staining of (**c**) Oatp4 and (**e**) Bsep was found in freshly isolated HSC, (**d, f**) but these proteins are up-regulated after TUDCA treatment. (**g**) The hepatocyte marker Ntcp was undetectable in freshly isolated HSC, (**h**) but was induced after TUDCA-initiated hepatic differentiation of HSC. The bile acid transporters Asbt, Oatp4, Bsep and Ntcp were co-stained with antibodies against desmin and vimentin (green). The low presence of Asbt in freshly isolated HSC could enable the cellular uptake of bile acids and their binding to Fxr. The increased expression of (**d**) Oatp4, (**f**) Bsep and (**h**) Ntcp confirmed the TUDCA-mediated hepatic differentiation of HSC.


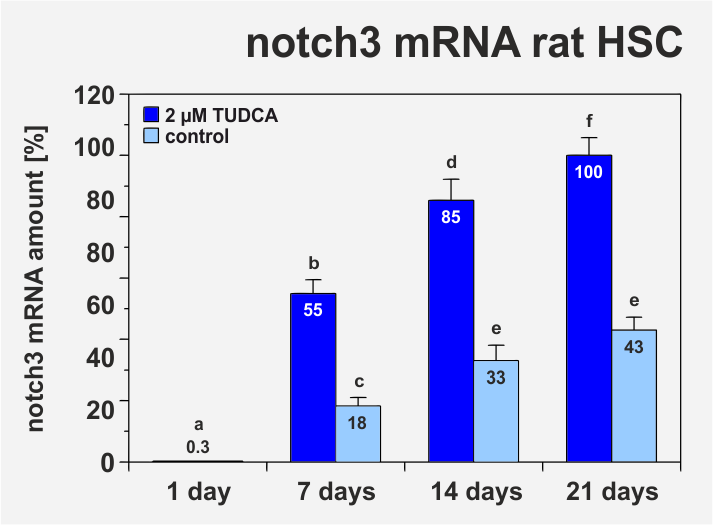


**Supplemental Figure S6|** **Elevated notch3 expression during TUDCA-mediated hepatic differentiation of rat HSC.** Freshly isolated HSC from rats were treated with 2 µM TUDCA for 21 days (n = 5). HSC of the control received the same medium but without TUDCA. The expression of the notch3 receptor was significantly up-regulated by TUDCA treatment compared to the control.


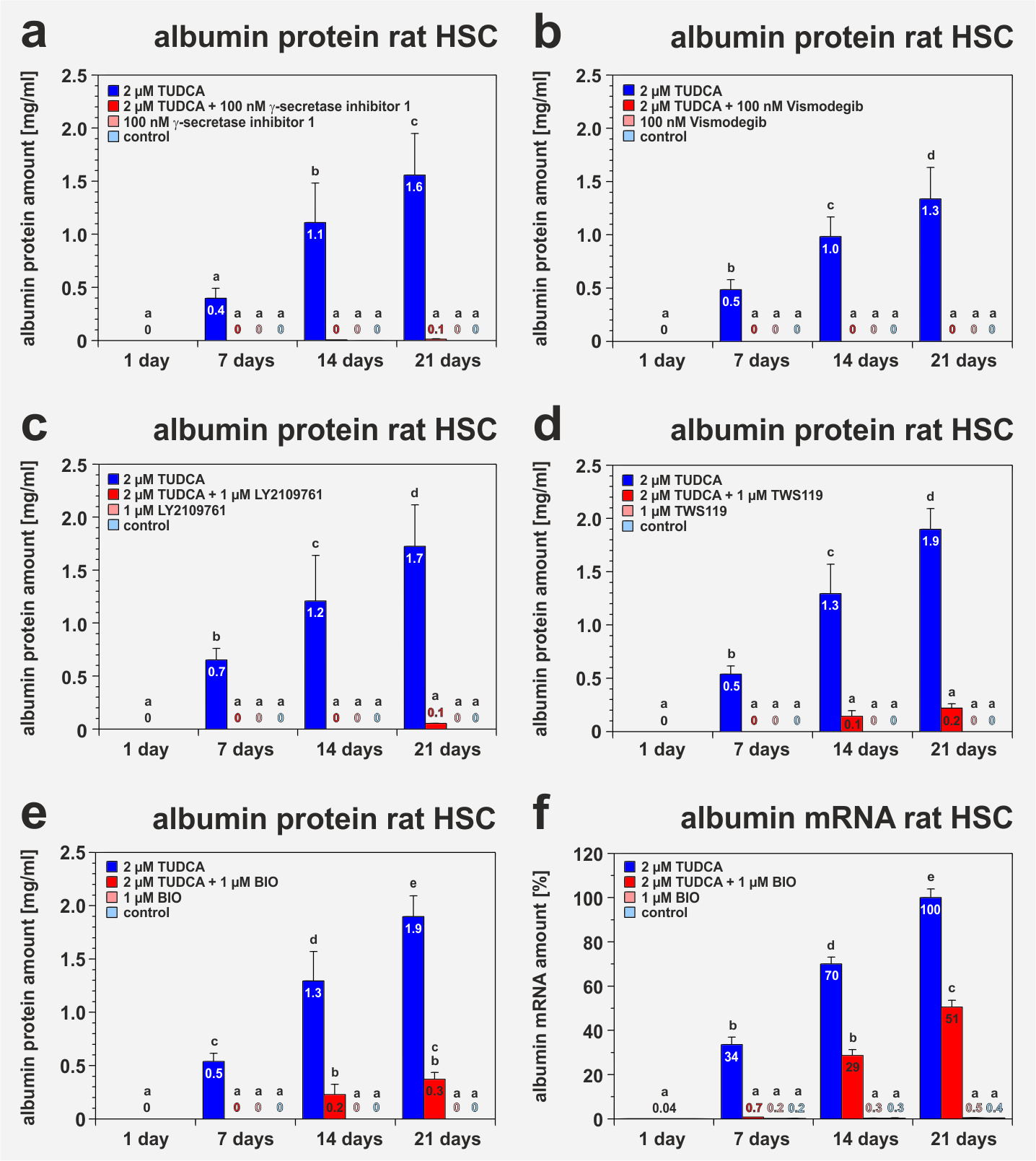


**Supplemental Figure S7|** **Notch, hedgehog, Tgf-β/Bmp and canonical Wnt signalling pathways control TUDCA-mediated hepatic differentiation of rat HSC.** Freshly isolated HSC from rats were treated with either 2 µM TUDCA, small inhibitory molecules or a combination of these substances for 21 days (n = 3). HSC of the control received medium without TUDCA and small inhibitory molecules. (**a-e**) The albumin protein concentration in the culture supernatants of HSC was investigated by an ELISA specific for rat albumin in weekly intervals (n = 3). (**a**) Notch signalling was inhibited by 100 nM γ-secretase inhibitor 1, which significantly blocked hepatic differentiation by TUDCA. Cell differentiation was also negatively affected when (**b**) hedgehog signalling was inhibited by 100 nM Vismodegib or (**c**) Tgf-β/Bmp signalling was inhibited by 1 µM LY2109761. Also the inhibition of Gsk3β activity by (**d**) 1 µM TWS119 or (**e**) 1 µM BIO, which mimic canonical Wnt signalling, prevented TUDCA-mediated hepatic differentiation of HSC as indicated by low albumin protein values. (**f**) The inhibitory effect of 1 µM BIO on albumin expression was also confirmed by qPCR.


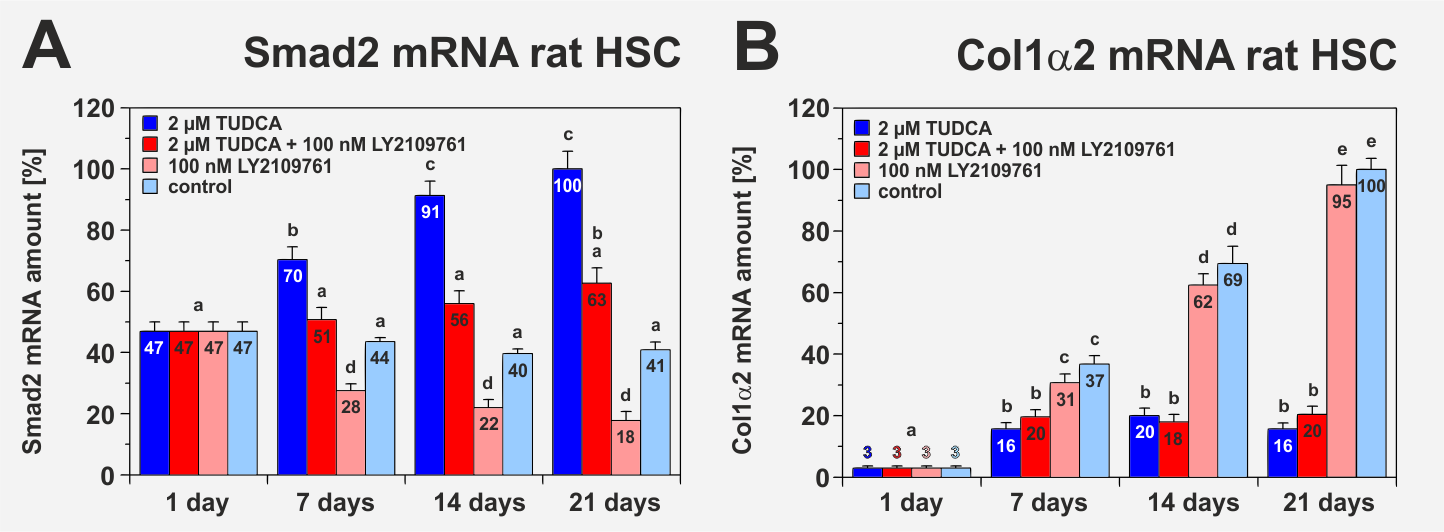


**Supplemental Figure S8|** **TUDCA-mediated hepatic differentiation of rat HSC involves Tgf-β/Bmp signalling.** Freshly isolated HSC from rats were treated with either 2 µM TUDCA, 1 µM LY2109761 (inhibitor of Tgf-β/Bmp signalling) or a combination of these substances for 21 days. HSC of the control received medium without TUDCA and LY2109761. (**a**) The expression of the Tgf-β/Bmp target gene Smad2 was investigated by qPCR in weekly intervals (n = 3). (**b**) The expression of collagen type 1 α2 chain (Col1α2) by HSC was analysed in the same experiments. (**a**) TUDCA treatment elevated and LY2109761 inhibited Smad2 expression in cultured HSC. (**b**) TUDCA-mediated hepatic differentiation decreased the expression of the fibrotic marker protein Col1α2, which was found to be regulated independently from Tgf-β/Bmp signalling.


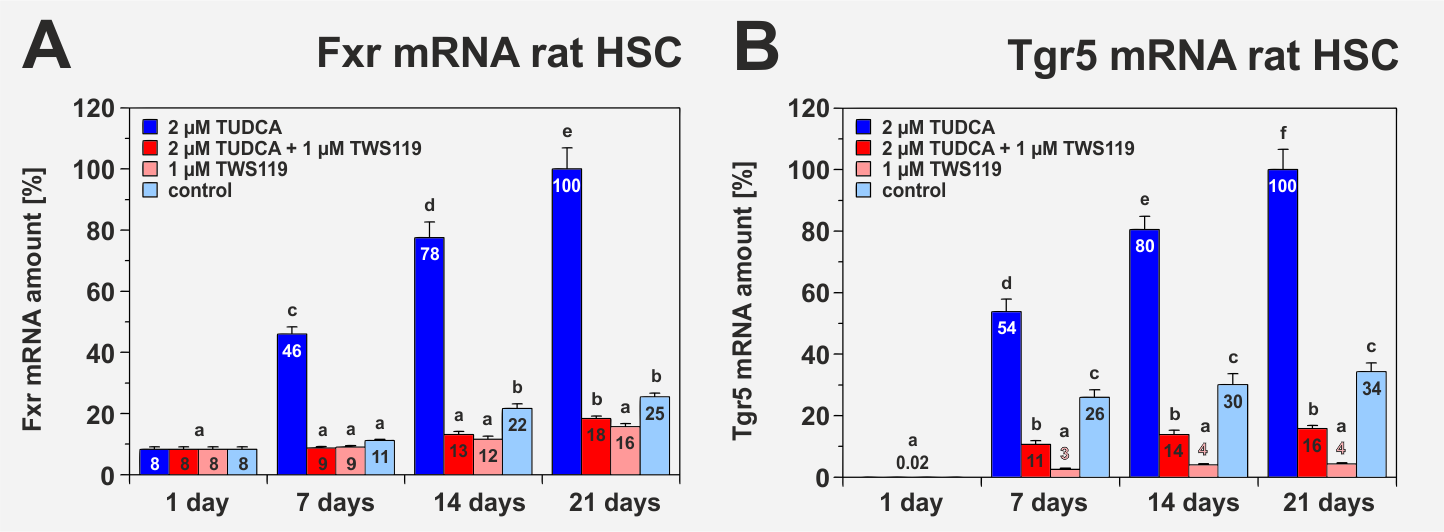


**Supplemental Figure S9|** **Canonical Wnt signalling inhibits TUDCA-mediated up-regulation of Fxr and Tgr5 expression in rat HSC.** Freshly isolated HSC from rats were treated with either 2 µM TUDCA, 1 µM TWS119 (Gsk3β inhibitor) or a combination of these substances for 21 days (n = 3). HSC of the control received medium without TUDCA and small inhibitory molecules. The expression of the bile acid receptors (**a**) Fxr and (**b**) Tgr5 was measured by qPCR in weekly intervals. TUDCA treatment enhanced Fxr and Tgr5 expression and this effect was abolished after mimicking of canonical Wnt signalling by TWS119 treatment.
